# MUC20 alleviates kidney fibrosis by modulating pyroptosis through the MET/RAS/STING axis

**DOI:** 10.7150/thno.123986

**Published:** 2026-02-04

**Authors:** Jiaxin Huang, Zhoutong Chen, Fengbo Zhong, Rui Zheng, Dexin Zhang, Jingyi Su, Yi Zhong, Xiaoliang Fang, Dali Li, Yuting Guan, Hongquan Geng

**Affiliations:** 1 Department of Urology, Children's Hospital of Fudan University, Shanghai, 201102, China.; 2 Shanghai Frontiers Science Center of Genome Editing and Cell Therapy, Shanghai Key Laboratory of Regulatory Biology, Institute of Biomedical Sciences and School of Life Sciences, East China Normal University, Shanghai, 200241, China.; 3 Department of Pediatric Urology, Xinhua Hospital Affiliated to Shanghai Jiao Tong University School of Medicine, Shanghai, 200092, China.

## Abstract

**Rationale:** Mucins are epithelial transmembrane glycoproteins involved in inflammation and kidney dysfunction, yet the role of the transmembrane mucin MUC20 in renal injury and fibrosis remains unclear. This study aimed to define the functional significance and underlying mechanisms of MUC20 in kidney fibrosis.

**Methods:** *Muc20*-deficient mice and tubular epithelial cell models were used to evaluate renal fibrosis and pyroptosis in induced kidney injury. Molecular and biochemical approaches were applied to assess protein interactions, RAS activation, 2′3′-cGAMP production, cGAS-STING signaling, lysosomal integrity, potassium efflux, and NLRP3 inflammasome activation.

**Results:** Loss of MUC20 significantly exacerbated renal fibrosis and increased pyroptosis in tubular epithelial cells. Mechanistically, MUC20 interacted with MET to promote RAS activation. MUC20 deficiency decreased GTP-bound RAS levels, leading to increased 2′3′-cGAMP production and activation of the cGAS-STING pathway. STING activation induced lysosomal membrane permeabilization, potassium efflux, and subsequent NLRP3 inflammasome-mediated pyroptosis.

**Conclusions:** MUC20 acts as a key protective regulator in kidney by restraining RAS-cGAS-STING-NLRP3-driven pyroptosis and fibrotic progression. Targeting MUC20-related signaling pathways may offer therapeutic potential for kidney fibrosis and chronic kidney disease.

## Introduction

Chronic kidney disease (CKD), a prominent global cause of morbidity and mortality, arises from congenital anomalies such as congenital anomalies of the kidney and urinary tract (CAKUT) or acquired injuries including ischemia, inflammation and obstruction. CKD often progresses to end-stage renal disease (ESRD), with a global prevalence ranging from 7% to 12% across regions^1^. Over the past 25 years, CKD has risen from the 25th to the 17th leading cause of death globally, which accounts for 1.35% of annual disability-adjusted life years (DALYs) and increasing by 1% yearly^2^, ^3^. Despite extensive research into CKD progression, its regulatory networks and underlying mechanisms remain incompletely understood and require further study^4^.

The mucin protein family includes transmembrane and secreted isoforms characterized by high molecular weight and extensive glycosylation. Recent studies highlight their roles in tumorigenesis, immune defense, and infection resistance^5^-^7^. Increasingly, mucins are recognized as key players in renal pathophysiology. For example, dysregulated *MUC1* expression drives autosomal dominant tubulointerstitial kidney disease (ADTKD)^8^-^10^. In acute kidney injury models, mucin 1 protects cells via HIF-1α and β-catenin pathway activation, influencing calcium channels^11^. *MUC4* and *MUC18* are linked to clear cell renal cell carcinoma and kidney stone formation^12^-^14^, with mucin 4 reducing oxidative stress and inhibiting calcium oxalate crystallization through ERK signaling^13^. Overall, these findings position mucins as promising therapeutic targets in kidney diseases.

*MUC20* encodes a cell surface-associated mucin predominantly expressed in epithelial tissues, contributing to barrier formation. Its C-terminus binds to MET, a multifunctional signaling platform, thereby activating downstream pathways^15^, ^16^. Beyond its physiological role, MUC20 is implicated in various diseases. An intracellular variant supports mitochondrial calcium balance and promotes chemoresistance in gastric cancer^17^. Overexpression predicts poor outcomes in endometrial cancer by enhancing EGF-induced malignancy via EGFR-STAT3 signaling^18^. In multiple myeloma, MUC20, regulated by extrachromosomal circular DNA (eccDNA), reduces proteasome inhibitor resistance through cuproptosis modulation^19^. In kidney disease, MUC20 was identified in human injured kidneys and is upregulated in IgA nephropathy, indicating its relevance to renal pathophysiology^20^, ^21^. However, its precise role in kidney injury and fibrosis remains unclear.

In this study, we show that MUC20 is a critical regulator of kidney injury and fibrogenesis. Our results demonstrate that *Muc20* deficiency exacerbates kidney damage and fibrosis in both unilateral ureteral obstruction (UUO) cisplatin-induced and folic acid (FA)-induced mouse models of CKD by promoting tubular epithelial cell death. Mechanistically, MUC20 interacts with MET to regulate RAS signaling. MUC20 deficiency leads to decrease in activated RAS (GTP-bound) and enhances 2'3'-cGAMP production, which activates the cGAS-STING axis. STING then triggers lysosomal membrane permeabilization, lysosome-induced cell death and potassium efflux, ultimately inducing NLRP3 inflammasome-mediated pyroptosis.

## Results

### Strong link between MUC20 and impaired kidney function

**Figure 1:**
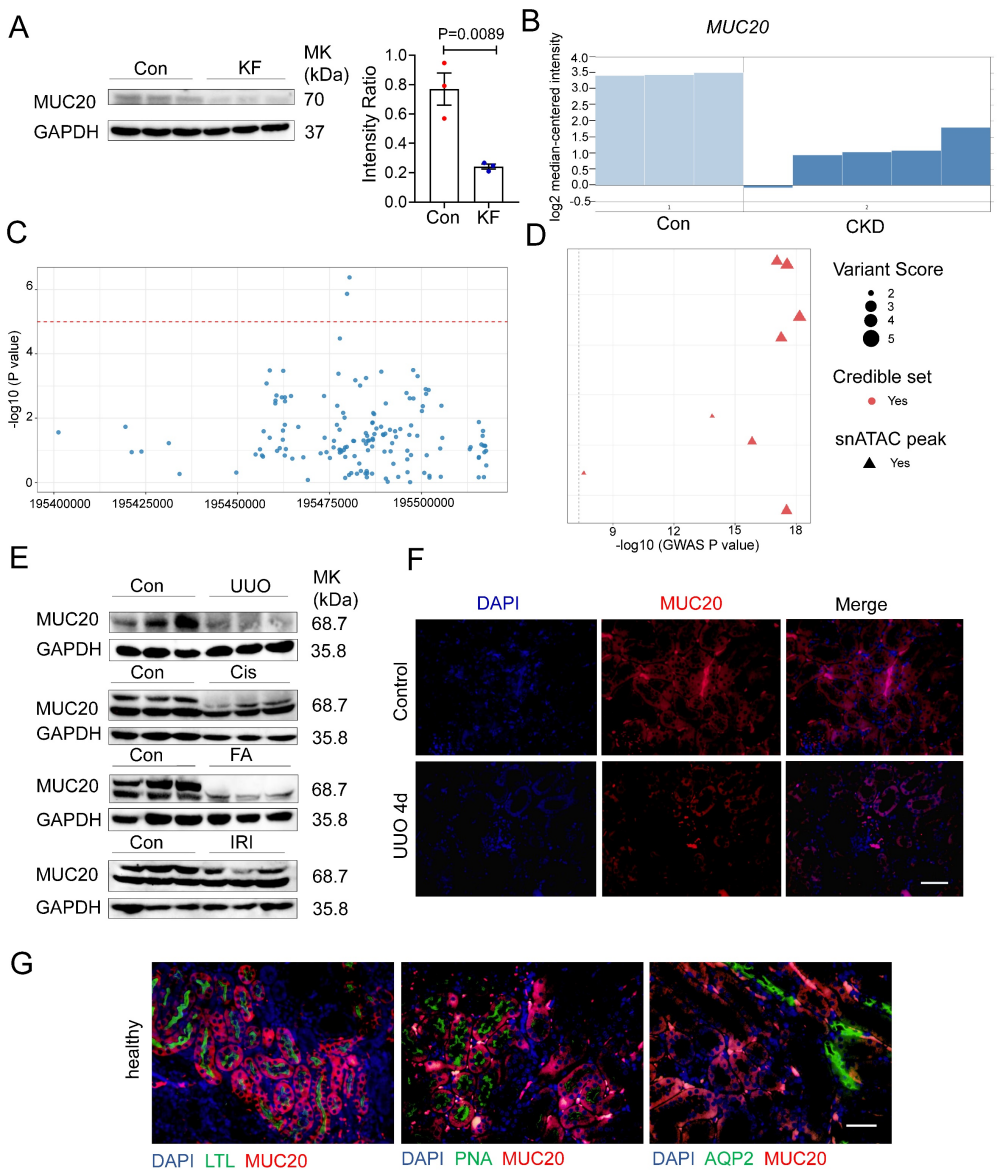
** Prominent correlation between MUC20 and impaired kidney function. A** Left: Western blots to detect protein level of MUC20 in kidney samples acquired from control groups and patients diagnosed with kidney fibrosis. Right: Ratio assessed by shade density compared with GAPDH as conference. **B** *MUC20* expression level in published Nakagawa CKD kidney data set. The presented chart was acquired from the validation set group compared to the normal kidney. Pale blue bars indicate MUC20 expression level in normal kidneys and dark blue bars indicate expression in the chronic kidney disease validation group samples. **C** Each point represents a single nucleotide polymorphism (SNP) within the genomic region encompassing the *MUC20* gene on chromosome 3 (GRCh37). The x-axis indicates the genomic position, and the y-axis shows the strength of association expressed as -log10(P value) from the CKDGen genome-wide association study for creatinine-based estimated glomerular filtration rate (eGFRcrea). The horizontal dashed red line denotes the nominal significance threshold (P = 1 × 10⁻⁵). Multiple variants within the *MUC20* locus display suggestive association signals, supporting the potential involvement of MUC20 in kidney function. **D** Each point represents a GWAS lead variant assigned to MUC20. The x-axis shows the strength of genetic association with kidney function (-log10 GWAS P value). Variants are colored by fine-mapping credible set membership and shaped by overlap with kidney single-nucleus ATAC-seq peaks. Point size reflects the integrated variant regulatory score. The dashed line indicates the genome-wide significance threshold (P = 5 × 10⁻⁸). **E** Western blots to detect protein levels of MUC20 in kidneys acquired from wild type mice following sham as the control group (n = 3), UUO-4-day surgery (n = 4), cisplatin peritoneal injection for 7 days (n = 3), folic acid (FA) intraperitoneal injection for 7 days (n = 3) and IRI surgery (n = 3). **F** Representative images of immunofluorescent staining of MUC20 in wild type mouse with or without UUO-induced kidney injury. Scale bar: 20μm. **G** Immunofluorescent double staining of MUC20 and kidney tubule specific markers including LTL, PNA and AQP2 for co-location detection in healthy wild type mouse kidney sections. Scale bar: 20 μm.

Given the known roles of mucins and mucin-domain transmembrane proteins in kidney disease^22^-^25^, we systematically evaluated mucin family members in mouse models of renal fibrosis. RT-qPCR revealed significant downregulation of *Muc20* in mice treated with FA **(Figure S1A)**. We further assessed MUC20 protein levels by western blot in kidney tissues from patients with fibrosis, revealing reduced expression compared to controls **(Figure 1A)**. Kidney function GWAS and eQTL data supported a key role for *MUC20* in fibrosis^26^, ^27^. Microarray analysis of renal biopsies from 48 CKD patients (discovery cohort) and 5 CKD patients (validation cohort) also showed lower *MUC20* expression in CKD samples^28^ **(Figure 1B)**. To evaluate the potential involvement of MUC20 in kidney function, we examined genome-wide association signals in the CKDGen consortium dataset for creatinine-based estimated glomerular filtration rate (eGFRcrea)^29^. Regional association analysis revealed multiple single nucleotide polymorphisms within and surrounding the *MUC20* locus showing clustered suggestive associations (P < 1 × 10⁻⁵) with eGFRcrea. Although no single variant reached genome-wide significance, the presence of multiple concordant association signals across the MUC20 region supports a potential contribution of this locus to kidney function** (Figure 1C)**. To further characterize genetic variants linked to *MUC20*, we then examined GWAS lead variants assigned to this gene and integrated association strength with regulatory annotations **(Figure S1B)**. Multiple lead variants displayed varying levels of association with kidney function, quantified by -log10 GWAS P values^30^. Fine-mapping analysis indicated that a subset of these variants belonged to credible sets, suggesting a higher probability of harboring causal signals. Notably, several variants overlapped with kidney single-nucleus ATAC-seq peaks, indicating localization within regions of open chromatin in kidney cells **(Figure 1D)**. Although only a limited number of variants surpassed the genome-wide significance threshold (P = 5 × 10⁻⁸), the convergence of association signals, fine-mapping support, and kidney-specific chromatin accessibility highlights *MUC20* as a genetically and functionally prioritized locus for kidney function. Genetic association investigation between genetic variations and serum creatinine based on Pan-UK Biobank highlights the *MUC20* locus as a region of interest for kidney-related traits and that genetic factors in or near this gene may influence creatinine homeostasis **(Figure S1C)**^31^. Moreover, *Muc20* expression was consistently reduced in UUO, cisplatin injection (Cis), FA and ischemia reperfusion injury (IRI) models compared to controls **(Figure 1E)**. Immunofluorescence confirmed decreased MUC20 levels in injured kidneys **(Figure 1F)**.

Among the mucin family, no significant changes were observed in other transmembrane (*Muc3*,* Muc13*,* Muc14*,* Muc15*,* Muc16*,* Muc21*) and secreted mucins (*Muc2*, *Muc5b*, *Muc6*) **(Figure S1D-E)***.* Previous studies localized MUC20 to tubule epithelial cells^20^. Using segmental markers (LTL, PNA, AQP2), we confirmed its predominant expression in proximal tubule epithelial cells **(Figure 1G)**, consistent with earlier findings. To investigate the potential role of MUC20 expression across different key cell types including stromal and immune compartments implicated in this process, we performed immunofluorescent double staining using MUC20 antibody together with lineage-specific markers—CD45 (pan-hematopoietic), CD68 and F4/80 (macrophages), and α-SMA (fibroblasts), and confirmed MUC20 is not expressed in these cells **(Figure S1F)**. Collectively, these findings suggest that *Muc20* is uniquely downregulated among mucins in injured kidneys.

### *Muc20* deficiency exacerbated renal injury in mice

To explore *Muc20*'s role in renal fibrosis, we generated *Muc20* knockout (KO) (*Muc20^-/-^*) mice using CRISPR/Cas9 targeting of exon 2 **(Figure S2A, Table S1)**. Sanger sequencing confirmed a 41-base pair deletion in the founder **(Figure S2B)**, resulting in significantly reduced *Muc20* transcript and protein levels compared to wide-type littermates **(Figure S2C-D)**, confirming successful knockout. Under basal conditions, no differences were observed in renal histology (H&E and Sirius Red staining) between genotypes **(Figure S2E)**. Blood urea nitrogen (BUN) and serum creatinine were similar in *Muc20^-/-^* mice and wildtype mice **(Figure S2F)**. Fibrosis extent demonstrated by *Col1a1*, *Col3a1*, *Fibronectin* and *Vimentin* transcription and renal segmental markers (*Nphs1*, Lrp2, *Umod*, *Slc12a3*, *Aqp2*,* Atp6v1g3*) also showed no significant differences **(Figure S2G-H)**. These results indicate that *Muc20^-/-^* mice have no detectable renal abnormalities under baseline conditions.

**Figure 2:**
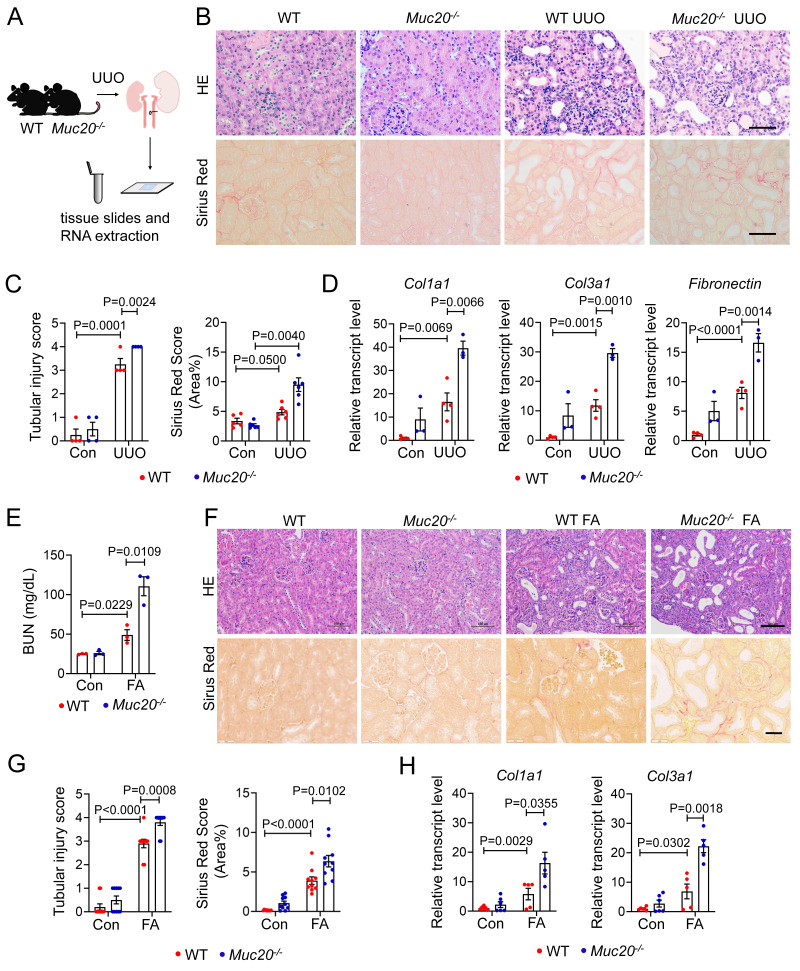
** Muc20 deficiency exacerbated renal injury in mice induced by UUO or folic acid. A** Flow chart and diagram of tissue slides and RNA extraction acquired from mice with KO and wild type genotypes induced by UUO surgery and sham surgery as the control group. **B** Kidney sections presented by HE-stained and Sirius Red-stained methods from experimental (WT n = 3, KO n = 4) and control groups (WT n = 4, KO n = 3) across two genotypes. Scale bar: 20 μm. **C** Left: scoring of renal tubular damage assessed by loss of the brush border, tubular dilation, inflammatory cell infiltration degree according to random HE-stained kidney cortical region sections. Right: scoring of Sirius Red staining assessed by percentage of red-stained collagen area in each selected random cortical region field. **D** Relative transcript level of fibrosis indicators including *Col1a1*, *Col3a1* and *Fibronectin* in UUO-4-day surgery treated wild type (n = 4) and *Muc20^-/-^* mice (n = 3) with sham-treated as control (WT n = 4, KO n = 3). **E** Serum blood urea nitrogen (BUN) measurement of wild type and *Muc20^-/-^* mice following sham (WT n = 3, KO n = 3) or FA intraperitoneal injection (WT n = 3, KO n = 3). Samples with hemolysis was excluded. **F** Kidney sections presented by HE-stained and Sirius Red-stained methods from folic acid intraperitoneal injection (WT n = 5, KO n = 5) and control groups (WT n = 6, KO n = 6) across two genotypes. Scale bar for HE-stained sections: 100 μm; scale bar for Sirius Red stained sections: 20 μm. **G** Left: Scoring of renal tubular damage assessed by loss of the brush border, tubular dilation, inflammatory cell infiltration degree according to random HE-stained kidney cortical region sections. Right: Scoring of Sirius Red staining assessed by percentage of red-stained collagen area in each selected random cortical region field. **H** Transcript level of fibrosis markers in kidneys of FA (WT n = 5, KO n = 5) and control groups (WT n = 6, KO n = 6) across two genotypes.

To assess* Muc20*'s pathophysiological role, we subjected age- and weight-matched wild-type and *Muc20^-/-^* littermates to 4-day UUO treatment **(Figure 2A)**, a well-established model of obstructive nephropathy^32^, ^33^. Histopathological analysis revealed more severe tubular dilation, increased inflammation, and greater collagen deposition in* Muc20^-/-^* mice after UUO, as shown by Sirius red staining **(Figure 2B-C)**. Consistently, expression of fibrosis-related genes (*Col1a1*, *Col3a1*, *Fibronectin*) was significantly upregulated in *Muc20^-/-^* kidneys **(Figure 2D)**. We validated these findings in the FA-induced nephropathy model, which mimics acute-to-chronic fibrotic progression^34^. After FA treatment,* Muc20^-/-^* mice exhibited elevated BUN levels, worse tubule dilation, and increased collagen accumulation versus controls **(Figure 2E-G)**. Transcriptional profiling further confirmed elevated expression of pro-fibrotic genes *Col1a1* and *Col3a1* in *Muc20^-/-^* mice **(Figure 2H)**. To test whether the exacerbated fibrotic phenotype persists in *Muc20^-/-^* mice within chronic models, we established an extended-duration model of kidney injury by administering cisplatin at a low dose (7 mg/kg) weekly for four weeks^35^. H&E and Sirius red staining of the chronic cisplatin-induced fibrosis model revealed a consistently exacerbated renal phenotype in KO mice, characterized by tubular dilation, immune cell infiltration, and increased collagen deposition **(Figure S3A)**. Quantitative analysis confirmed a significant increase in fibrosis **(Figure S3B)**. In line with the aggravated histological damage, cisplatin-treated KO mice exhibited markedly higher serum creatinine levels than WT counterparts **(Figure S3C)**. Furthermore, the expression of key renal fibrosis markers *Col1a1, Col3a1, Fibronectin* and *Vimentin* was further upregulated in KO kidneys, corroborating the enhanced fibrotic response **(Figure S3D)**. These results suggest that *Muc20* deficiency exacerbated renal injury in mouse models.

### RNA sequencing shows increased cell death in *Muc*20-deficient conditions

**Figure 3:**
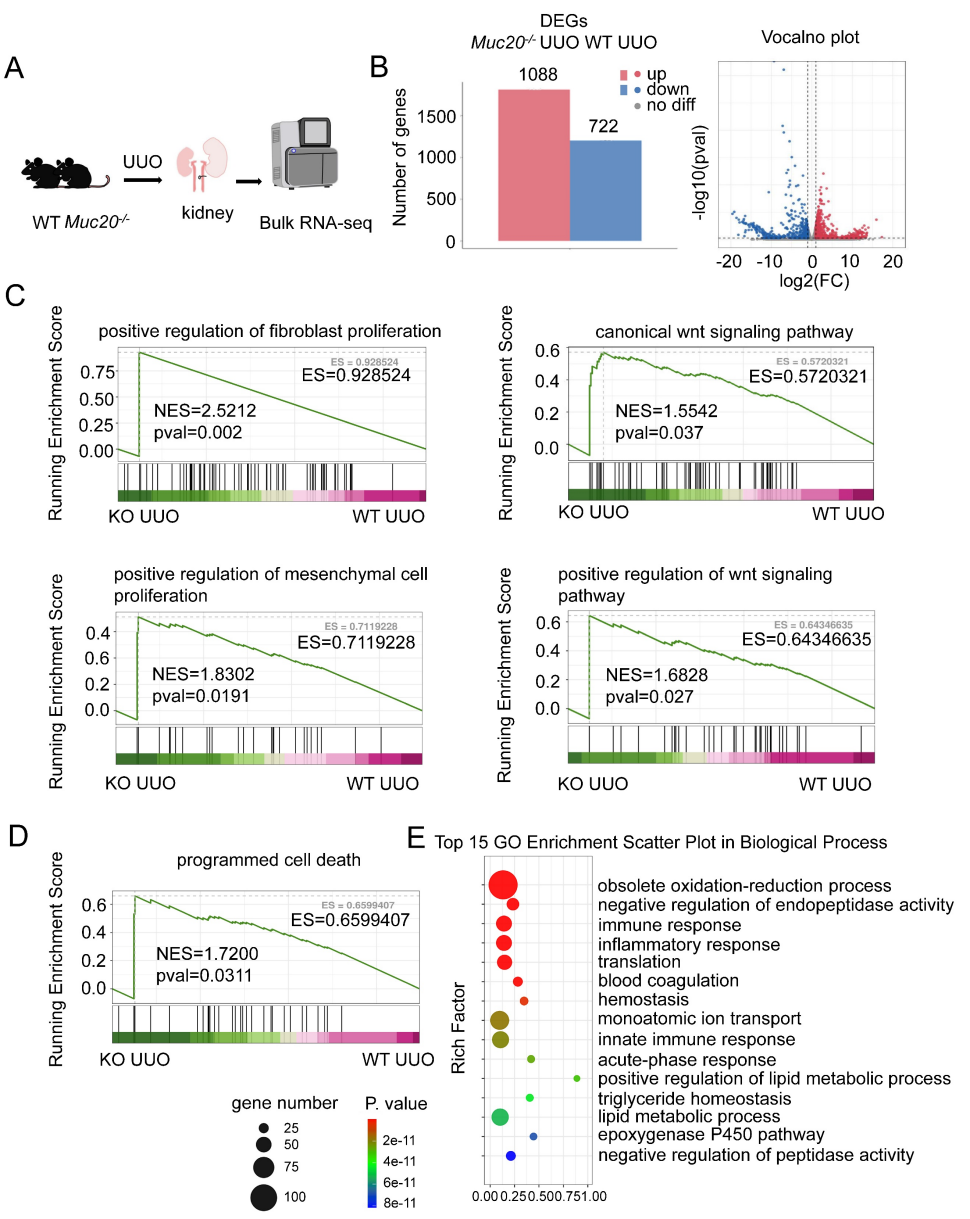
** MUC20 regulates cellular death after induced kidney impairment. A** Flow chart and diagram of the bulk RNA-seq procedure for kidney treated with UUO surgery. **B** Left: Bar plots of differential expressed up-regulated and down-regulated genes according to bulk RNA-seq analysis results within two genotypes. Right: Volcano plots of differential expressed up-regulated and down-regulated genes according to bulk RNA-seq analysis. Red bars represent up-regulated gene number and blue bars represent down-regulated gene number. **C** GSEA analysis of fibrosis related procedures including positive regulation of fibroblast proliferation, canonical Wnt signaling pathway, positive regulation of mesenchymal cell proliferation and positive regulation of Wnt signaling pathway. **D** GSEA analysis of programmed cell death from two genotypes. **E** Top15 GO enrichment analysis of WT and *Muc20* KO kidneys after treatment with UUO surgery from bulk RNA-seq data.

We performed bulk RNA sequencing on kidneys from wildtype and *Muc20^-/-^* mice after UUO treatment **(Figure 3A)**. Comparative analysis identified 1810 differentially expressed genes (DEGs) in *Muc20^-/-^* mice, with 1088 upregulated and 722 downregulated transcripts **(Figure 3B)**. Gene set enrichment analysis (GSEA) revealed significant activation of fibrosis- related pathways, including regulation of fibroblast proliferation, mesenchymal cell proliferation, and Wnt signaling, pathways known to drive renal injury and fibrosis^36^-^38^. This gene expression pattern aligns with histological evidence of worsened fibrosis in *Muc20^-/-^* mice **(Figure 3C)**. Notably, GSEA and GO enrichment also showed elevated immune and inflammatory responses, as well as programmed cell death pathways, suggesting that increased cell death contributes to the *Muc20* KO phenotype **(Figure 3D-E)**. Collectively, these findings indicate that loss of MUC20 aggravates kidney injury and promotes fibrotic progression in mouse models of both obstructive and toxin-induced nephropathy.

### Deficiency in MUC20 leads to increased cell mortality

**Figure 4:**
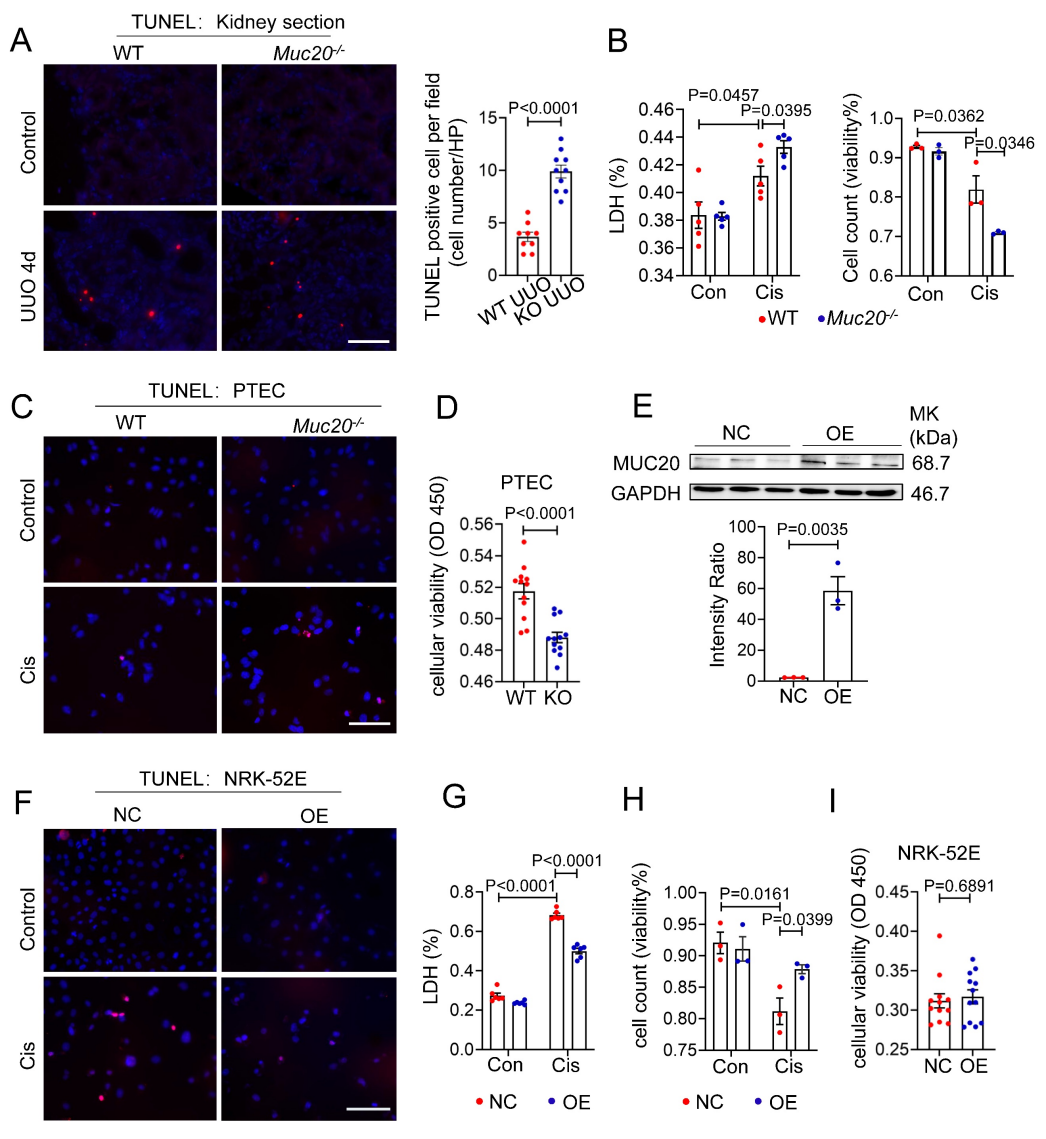
** Deficiency in MUC20 induces severer cell mortality. A** Left: Representative TUNEL assay of kidney sections acquired from wild type and *Muc20^-/-^* mice following sham or UUO-4-day surgery treatment. Right: Quantification of TUNEL positive cells within each selected field (n = 10). Scale bar: 20 μm. **B** Left: LDH level of primary tubular epithelial cell acquired from 3-week-old WT and *Muc20^-/-^* mice following sham or Cisplatin treatment. Right: Viability of primary tubular epithelial cell acquired from 3-week-old WT and *Muc20^-/-^* mice following sham or cisplatin treatment assessed by Trypan Blue staining assay with microscopic direct cytometry. **C** Representative TUNEL assay of primary tubular epithelial cells acquired from 3-week-old WT and *Muc20^-/-^* mice following sham or cisplatin treatment. Scale bar: 20 μm. **D** Viability of primary tubular epithelial cell acquired from 3-week-old WT and *Muc20^-/-^* mice following Cisplatin treatment for 16 h assessed through CCK8 Assay and evaluated by OD value at 450nm absorption spectrum. **E** Up: Western blots for protein level detection of MUC20 in stable MUC20 over-expressed transfected NRK-52E cell line (OE) and sham-vector transfected NRK-52 cells as negative control (NC). Down: Ratio assessed by shade density compared with conference GAPDH. **F** Representative TUNEL assay of NC cell and OE cell challenged with cisplatin compared to control group. Scale bar: 20 μm. **G** LDH releasing detection NC cell and OE cell after cisplatin challenge.** H** Viability of NC cell and OE cell following sham or cisplatin treatment assessed via Trypan Blue staining assay with microscopic direct cytometry.** I** Viability of NC cell and OE cell following cisplatin treatment for 16 h assessed through CCK8 Assay and evaluated by OD value at 450nm absorption spectrum.

Given that RNA sequencing revealed signs of programmed cell death in UUO-treated *Muc20^-/-^* kidneys, we further evaluated this phenotype in kidney sections. Histological analysis showed a significant increase in TUNEL positive puncta in *Muc20^-/-^* mice compared to controls **(Figure 4A)**, suggesting enhanced apoptotic or apoptosis-like activity in the absence of *Muc20.* To validate this *in vitro*, we treated primary tubular epithelial cells (PTECs) from both genotypes with 20 μM cisplatin, a standard model of nephrotoxicity^39^. After 24 hours, *Muc20^-/-^* PTECs exhibited markedly elevated cytotoxicity, as evidenced by higher lactate dehydrogenase (LDH) release, fewer viable cells by trypan blue staining, and more TUNEL positive puncta **(Figure 4B-C)**. Consistent with these results, CCK-8 assays confirmed reduced cell viability in *Muc20^-/-^* PTECs versus wild-type controls **(Figure 4D)**, supporting a key role for *Muc20* in promoting cell survival under stress.

To further explore MUC20's cytoprotective function, we generated *Muc20-*overexpressing NRK-52E rat renal tubular cells (OE) **(Figure 4E)**. Notably, *Muc20* overexpression significantly reduced cisplatin-induced damage, as evidenced by fewer TUNEL positive cells and lower LDH release **(Figure 4F-G)**. In addition, both trypan blue exclusion and CCK-8 assays showed improved cell viability in OE cells **(Figure 4H-I)**. Collectively, these *in vitro* and *in vivo* findings demonstrate that MUC20 strongly protects renal tubular epithelial cells against cisplatin-induced cytotoxicity and cell death.

To explore the mechanism by which MUC20 regulates cell death, we assessed key pathways, apoptosis, necroptosis and ferroptosis, in UUO-treated kidneys and in *Muc20* KO or overexpressing cells exposed to cisplatin. For apoptosis, *Bax* and* Bak1* expression increased in response to UUO or cisplatin treatment, but no significant differences were observed between WT, *Muc20* KO or OE conditions **(Figure S4A)**. Similarly, transcript levels of *Ripk1* and *Ripk3*, markers of necroptosis, were elevated after injury but showed no genotype-dependent variation **(Figure S4B)**. No differences in the ferroptosis-related gene *Acsl4* were detected between WT and KO kidneys post UUO **(Figure S4C)**. Annexin V-mCherry/SYTOX green staining further confirmed no significant differences in necrotic and apoptotic cell levels between genotypes following cisplatin treatment **(Figure S4D)**. Taken together, these data demonstrate that *Muc20* deficiency exacerbates renal injury through a mechanism independent of classical apoptosis, necroptosis or ferroptosis, suggesting an alternative cytoprotective role for MUC20.

### Deficiency in MUC20 activates pyroptosis during injury

**Figure 5:**
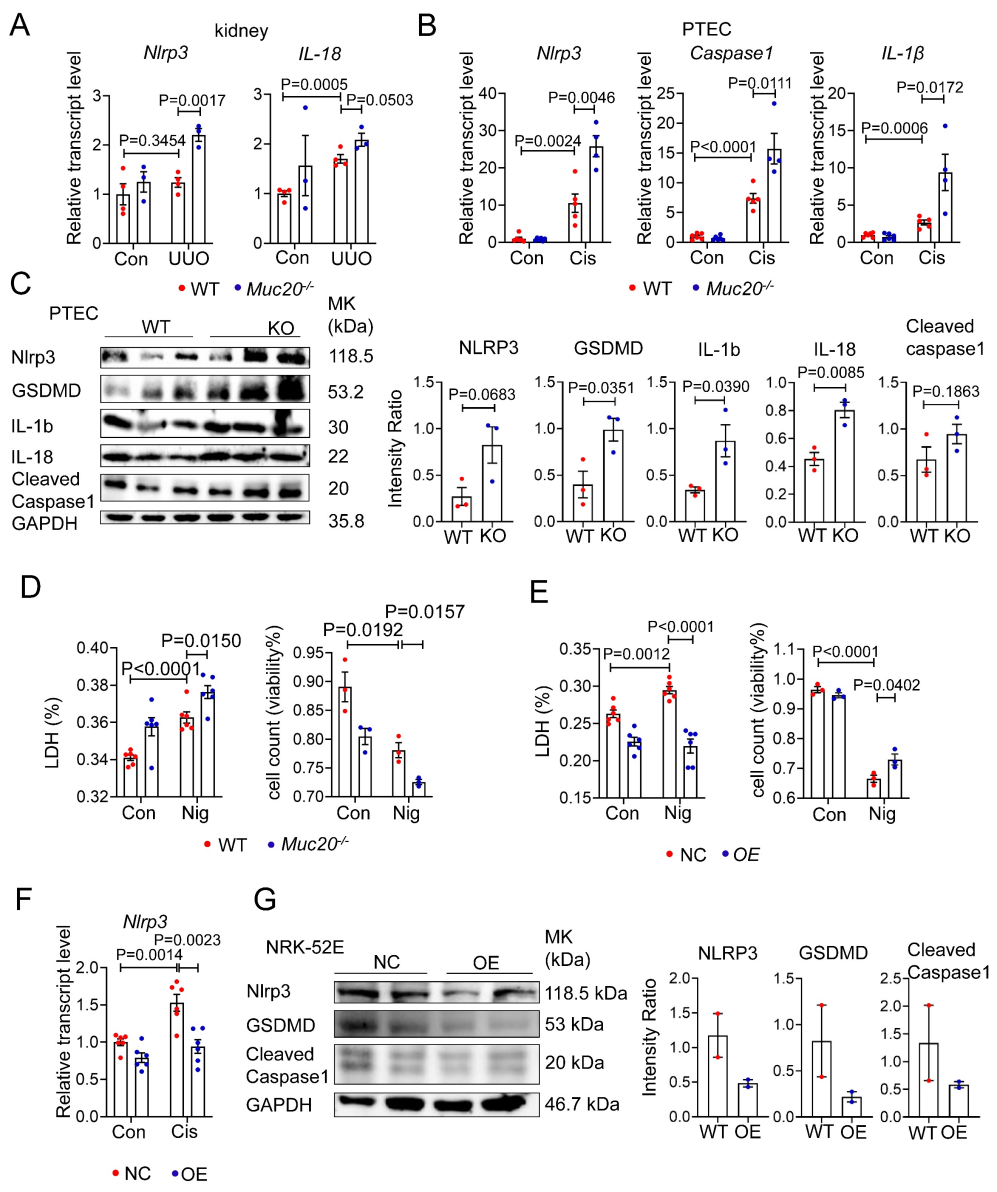
**Deficiency in MUC20 activated pyroptosis during injury. A** Relative transcript level of pyroptosis markers protein level *Nlrp3*, *IL-18* in kidneys of UUO-4-day surgery treated wild type (n = 4) and *Muc20^-/-^* mice (n = 3) with sham-treated as control (WT n = 4, KO n = 3). **B** Pyroptosis markers including *Nlrp3*, *Caspase-1* and *IL-1β* transcriptive detection in primary tubular epithelial cell with cisplatin challenge. **C** Left: Western blots for pyroptosis related markers protein level detection in primary tubular epithelial cell acquired from 3-week-old WT and *Muc20^-/-^* mice at basal line. Right: Quantified analysis about NLRP3, GSDMD, IL-1b, IL-18 and cleaved caspase 1 compared with GAPDH within WT and KO mice at basal line. **D** Left: LDH level of primary tubular epithelial cell acquired from 3-week-old WT and *Muc20^-/-^* mice following sham or Nigericin treatment. Right: Viability of primary tubular epithelial cell acquired from 3-week-old WT and *Muc20^-/-^* mice following sham or Nigericin treatment assessed via Trypan Blue staining assay with microscopic direct cytometry. **E** Left: LDH level of NC cell and OE cell following sham or Nigericin treatment. Right: Viability of NC cell and OE cell following sham or Nigericin treatment assessed via Typran Blue staining assay with microscopic direct cytometry. **F** Relative transcript level of pyroptosis markers *Nlrp3* in NC cell and OE cell at basal line and after treatment with Cisplatin for 16 hours. **G** Western blots for pyroptosis related markers protein level detection in NC cell and OE cell at basal line.

To determine whether MUC20 modulates pyroptosis, we analyzed key pyroptotic markers in *Muc20* KO kidneys and cells under injury conditions. qPCR revealed significant upregulation of *Nlrp3*, *Caspase 1* and* Il-1β* in injured *Muc20* KO kidneys and cells compared to WT controls **(Figure 5A-B)**. Western blot analysis confirmed elevated expression of pyroptotic proteins including GSDMD, IL-1β, IL-18, NLRP3 and cleaved Caspase1 as well as cleaved N-terminal GSDMD (N-GSDMD) **(Figure 5C, Figure S5A)**, indicating enhanced pyroptotic activity in *Muc20*-deficient cells.

To further assess MUC20's role in pyroptosis, we treated WT and KO PTEC with the pyroptosis inducer Nigericin. *Muc20^-/-^* cells exhibited significantly increased nigericin-induced cell death **(Figure 5D)**, whereas *Muc20* overexpressing cells exhibited attenuated pyroptotic injury **(Figure 5E)**. Furthermore, *Muc20* overexpression suppressed baseline and cisplatin induced *Nlrp3* expression as well as cleaved N-terminal GSDMD at basal line **(Figure 5F-G, Figure S5B)**. In summary, our findings demonstrate that *Muc20* acts as a critical suppressor of pyroptosis, protecting tubular epithelial cells by suppressing NLRP3 inflammasome activation and pyroptotic cell death.

Plasma membrane rupture and increased membrane permeability are hallmark structural features of pyroptotic cell death. To further substantiate the structural alterations associated with pyroptosis, we performed propidium iodide (PI) staining on cisplatin-treated primary renal epithelial cells isolated from wild-type (WT) and *Muc20*-KO mice, followed by flow cytometric quantification of PI-positive cells. Under identical cisplatin stimulation conditions, KO epithelial cells exhibited a significantly higher proportion of PI-positive cells, indicating enhanced membrane permeability and increased susceptibility to pyroptotic cell death **(Figure S5C-D)**.

Consistent with these findings, parallel experiments conducted in NRK-52E cells overexpressing *Muc20* demonstrated a marked reduction in the proportion of PI-positive cells. Both confocal microscopy and flow cytometric analyses corroborated that MUC20 overexpression attenuates cisplatin-induced membrane permeabilization, further supporting a protective role of MUC20 against pyroptosis-associated structural damage **(Figure S5E-F)**.

### Pharmacological inhibition of pyroptosis reduces renal injury and fibrosis in *Muc20* KO mice

**Figure 6:**
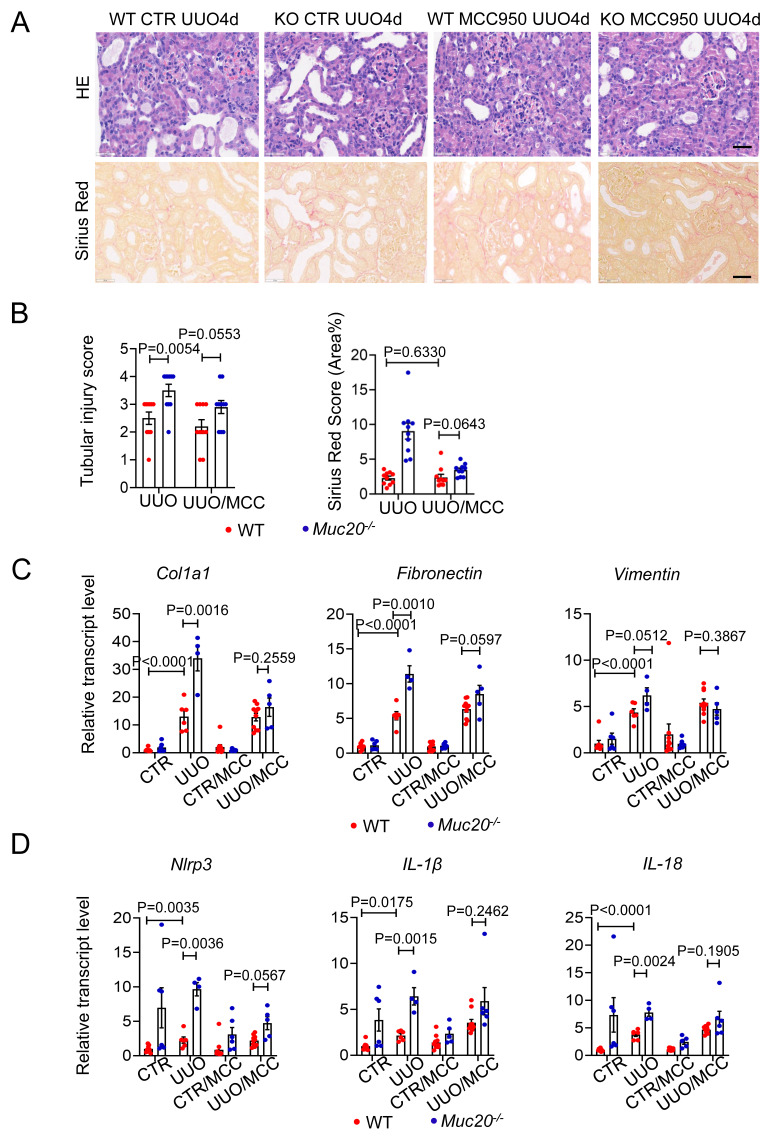
**Pharmacological inhibition of pyroptosis attenuated injury and fibrosis extent of UUO induced kidney fibrosis. A** Kidney sections treated with HE-staining and Sirius Red-staining from mice treated with sham (WT n = 8, KO n = 6), sham with antagonist treatment (WT n = 10, KO n = 6), UUO-4-day surgery treatment (WT n = 6, KO n = 4) and UUO-4-day surgery with antagonist (WT n = 10, KO n = 5) across genotypes. Scale bar: 20 μm. **B** Scoring of renal tubular damage assessed by pathological features according to random HE-stained kidney cortical region sections. **C** Relative transcript level of fibrosis indicators including *Col1a1*, *Fibronectin*, *Vimentin* in UUO-4-day surgery treated wild type and *Muc20^-/-^* mice with or without antagonist injection and sham-treated as control. **D** Relative transcript level of pyroptosis markers *Nlrp3*,* Caspase 1*, *IL-1β* and *IL-18* in kidneys of UUO-4-day surgery treated wild type and *Muc20^-/-^* mice with (WT n = 10, KO n = 5) or without antagonist injection (WT n = 6, KO n = 4) and sham-treated as control (WT n = 8, KO n = 6; with antagonist WT n = 10, KO n = 6).

To determine whether NLRP3-mediated pyroptosis contributes to the exacerbated renal injury in *Muc20* KO mice. We treated both WT and *Muc20* KO mice with the selective NLRP3 antagonist MCC950 before UUO surgery. MCC950 significantly reduced renal damage in *Muc20* KO mice compared to WT mice, as evidenced by decreased tubular dilation and immune cell infiltration **(Figure 6A-B)**. Consistent with these results, transcriptional analysis showed downregulation of fibrotic markers (*Col1a1*,* Fibronectin*,* Vimentin*) in *Muc20^-/-^* kidneys after MCC950 treatment **(Figure 6C)**. In addition, MCC950 inhibited the NLRP3 inflammasome pathway in *Muc20* KO kidneys, as demonstrated by reduced transcriptional expression of *Nlrp3*, *IL-1β* and *IL-18* **(Figure 6D)**. These results show that NLRP3-dependent pyroptosis largely drives the enhanced renal injury in *Muc20*-deficient mice, and its pharmacological inhibition effectively alleviates both tissue damage and fibrotic progression.

### MUC20 modulates pyroptosis through lysosomal-STING-potassium efflux signaling

**Figure 7:**
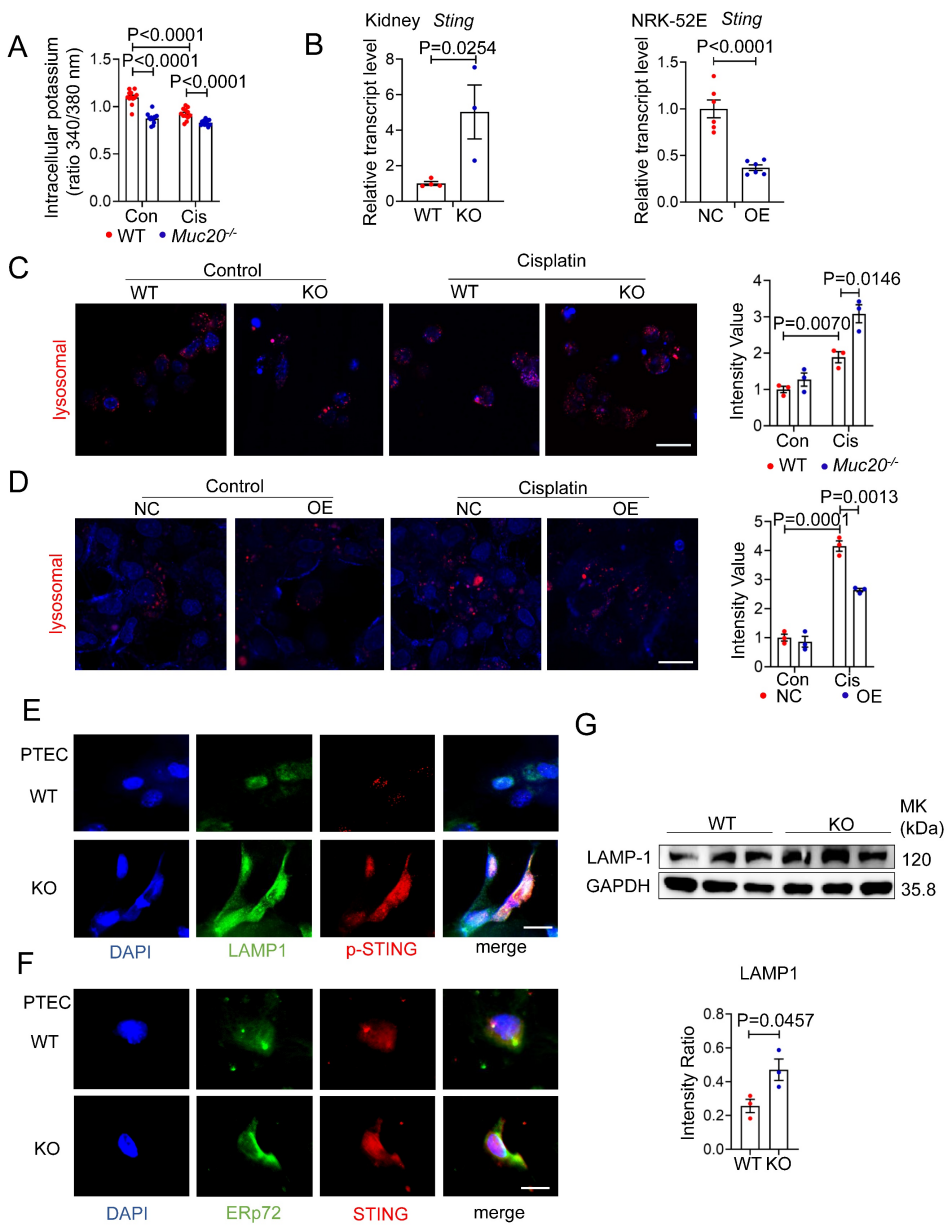
**MUC20 modulated pyroptosis through lysosomal-STING-potassium efflux signaling. A** Detection of intracellular potassium in primary tubular epithelial cell acquired from 3-week-old WT and *Muc20^-/-^* mice following sham or Cisplatin treatment for 16 hours. The data was presented as the form of fluorescence signal ratio acquired from Ex (excitation Wavelength) at 340nm by Ex at 380nm, Em (Emission Wavelength) at 500nm. **B** Left: Relative transcript level of *Sting* in kidneys of UUO-4-day surgery treated wild type and *Muc20^-/-^* (WT n = 4, KO n = 3) and sham-treated as control group (WT n = 4, KO n = 3). Right: Relative transcript level of *Sting* in NC cell and OE cell following treatment with Cisplatin for 16 hours (NC n = 6, OE n = 6) or sham as control (NC n = 6, OE n = 6). **C** Left: Detection of lysosomal in primary tubular epithelial cell acquired from 3-week-old WT and *Muc20^-/-^* mice following sham or Cisplatin treatment for 16 hours through Lyso-Track Red assay under confocal microscope. Red signal dots indicate stained lysosomal. Right: Statistical analysis of stained lysosomal intensity in each three fields from WT and KO groups with Cisplatin treatment or sham as control group. Scale bars: 20μm. **D** Left: Detection of lysosomal in NC cell and OE cell following sham or Cisplatin treatment for 16 hours through Lyso-Track Red assay under confocal microscope. Red signal dots indicate stained lysosomal. Right: Statistical analysis of stained lysosomal intensity in each three fields from WT and KO groups with Cisplatin treatment or sham as control group. Scale bars: 20μm.** E** Co-location detection from immunofluorescent staining of ERp72 and STING in primary tubular epithelial cell acquired from 3-week-old WT and *Muc20^-/-^* mice without treatment at basal line. Scale bar: 10μm.** F** Co-location detection from immunofluorescent staining of LAMP-1 and p-STING in primary tubular epithelial cell acquired from 3-week-old WT and *Muc20^-/-^* mice without treatment at basal line. Scale bar: 10μm. **G** Western blots for protein level detection of pyroptosis related markers in primary tubular epithelial cell acquired and cultured from 3-week-old WT and *Muc20^-/-^* mice.

Having established MUC20's protective role against pyroptosis mediated kidney injury, we investigated its underlying mechanism. Since potassium efflux is a key trigger for NLRP3 inflammasome activation^40^, we measured intracellular potassium levels in cisplatin treated WT and *Muc20* KO cells. Notably, *Muc20* KO cells showed significantly lower potassium levels at baseline and after cisplatin treatment **(Figure 7A)**, suggesting heightened NLRP3 activation in the absence of MUC20.

Recent studies link cGAS-STING signaling and lysosomal membrane permeabilization to pyroptosis through potassium efflux^40^-^43^. Therefore, we examined STING and lysosome activity. *Muc20* KO kidneys exhibited elevated *Sting* mRNA expression compared to WT controls, while *Muc20* overexpression suppressed *Sting* levels **(Figure 7B)**. LysoTracker staining revealed increased lysosomal accumulation in *Muc20^-/-^* cells under basal and cisplatin treated conditions **(Figure 7C)**, whereas *Muc20* overexpression in NRK-52E cells reduced lysosomal aggregation **(Figure 7D)**. We further assessed lysosome and cGAS-STING activation in PTECs. Immunofluorescence showed that inactive STING localized similarly to the endoplasmic reticulum (ER) (marked by ERp72) in both WT and KO cells, However, phosphorylated STING (p-STING) was markedly elevated in *Muc20* KO cells **(Figure 7E-F)**. Importantly, p-STING in KO cells co-localized predominantly with lysosomes (marked by LAMP1), rather than ER, consistent with known ER-to-lysosome trafficking pathways^44^. Western blot analysis confirmed higher LAMP-1 levels in *Muc20*-deficient cells **(Figure 7G)**.

Previous studies have suggested that ER stress represents an important upstream trigger for STING activation and that activation of the STING pathway can subsequently drive interferon production and antiviral immune responses^45^, ^46^. To evaluate whether ER stress contributes to STING activation in our model, we examined the expression of key ER stress associated mediators in kidneys from WT and *Muc20^-/-^* mice subjected to UUO induced fibrosis. Notably, the expression levels of canonical ER stress markers, including* Chop*, *Ern1*, *(Ire1α)*, *Perk*, and *Atf6*, were comparable between WT and KO kidneys following UUO **(Figure S5G)**. In parallel, we assessed the expression of representative STING downstream genes associated with interferon signaling and immune activation, including *Isg15*, *Cxcl10*, and *Ifnb1*, and observed no significant differences between WT and KO tissues **(Figure S5H)**. Collectively, these findings indicate that ER stress is unlikely to be the primary driver of STING activation in this context and further suggest that STING signaling preferentially associated with interferon production is not engaged in the UUO model examined here.

In summary, our findings show that MUC20 deficiency promotes NLRP3-dependent pyroptosis via the STING-lysosomal-potassium efflux axis. Loss of MUC20 activates STING, which traffics to lysosomes, triggering potassium efflux and promoting NLRP3 inflammasome assembly. This pathway reveals a novel mechanism underlying MUC20's cytoprotective function in renal epithelial cells.

### MUC20 regulates the STING pathway via direct interaction with MET and GTP homeostasis

**Figure 8:**
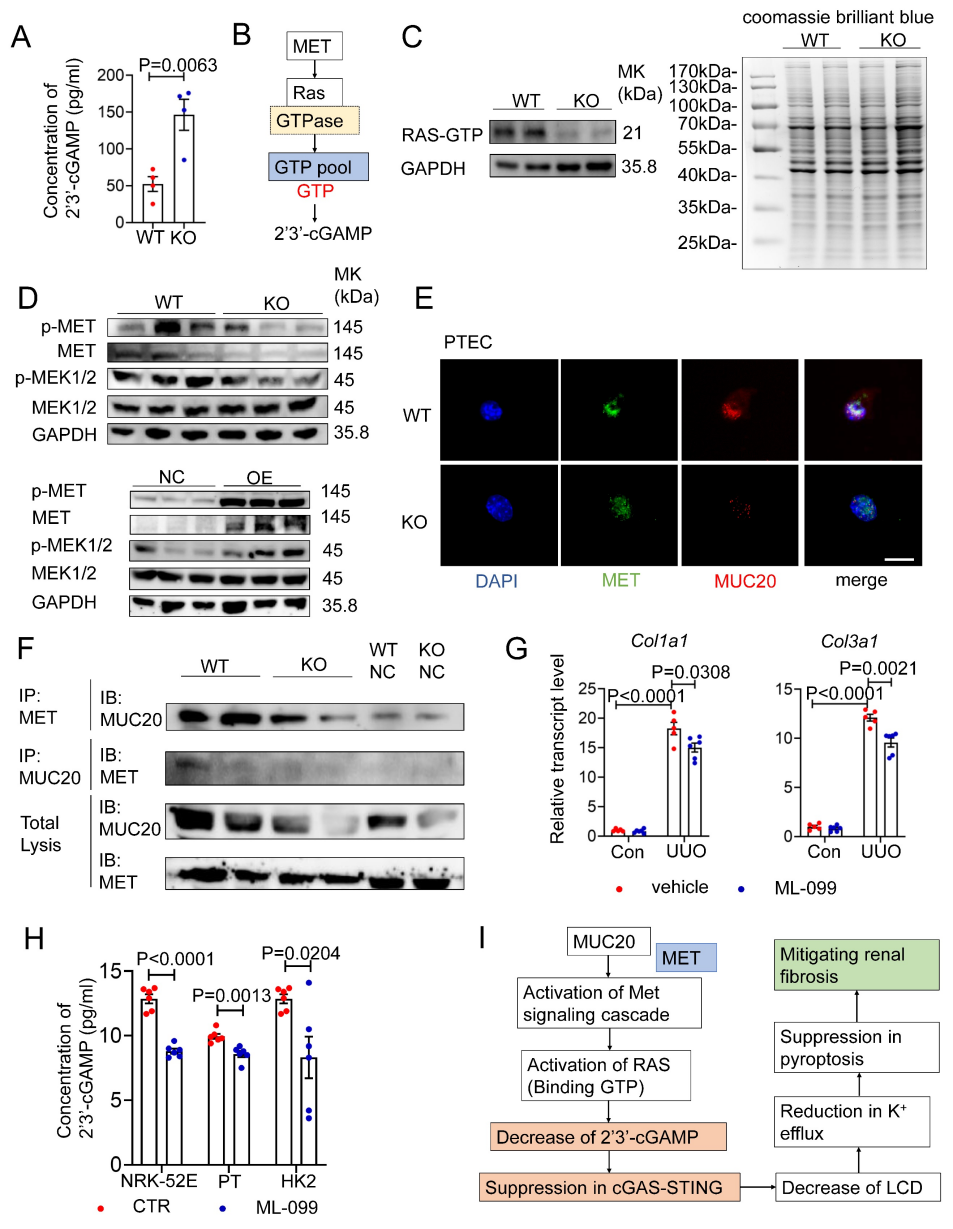
** MUC20 interferes STING cascade through interacting with MET directly and GTP homeostasis regulation. A** Detection of concentration of intracellular 2'3'-cGAMP in primary tubular epithelial cell without treatment at basal line acquired and cultured from 3-week-old WT and *Muc20^-/-^* mice by ELISA assay. **B** Pipeline of cascade about MET influencing synthesis of 2'3'-cGAMP via affecting GTPase Ras.** C** Left: RAS-GTP pull down assay for detection of activated RAS in primary tubular epithelial cell without treatment at basal line acquired and cultured from 3-week-old WT and *Muc20^-/-^* mice. Right: Coomassie Blue Staining for whole protein level detection for primary tubular epithelial cell without treatment at basal line acquired and cultured from 3-week-old WT and *Muc20^-/-^* mice. **D** Up: Western blots for protein level detection of MET/MEK signaling cascade related markers in primary tubular epithelial cell acquired and cultured from 3-week-old WT and *Muc20^-/-^* mice without treatment at basal line. Down: Western blots for protein level detection of MET/MEK signaling cascade related markers in NC and OE cell without treatment at basal line. **E** Co-location detection from immunofluorescent staining of MET and MUC20 in primary tubular epithelial cell acquired from 3-week-old WT and *Muc20^-/-^* mice without treatment at basal line. Scale bar: 10μm. **F** Co-IP assay to detect interaction between MUC20 and MET in primary tubular epithelial cell acquired from 3-week-old WT and *Muc20^-/-^* mice without treatment at basal line. The upper row indicates MET-antibody coated beads and the western blot assay with MUC20 antibody. **G** Relative transcript level of *Col1a1* and *Col3a1* in kidneys of UUO-4-day surgery treated wild type with (sham n = 5, UUO n = 5) or without (sham n = 6, UUO n = 6) RAS related GTPases agonist ML-099 injection and sham-treated as control group. **H** Detection of concentration of intracellular 2'3'-cGAMP in primary tubular epithelial cell acquired and cultured from 3-week-old WT and *Muc20^-/-^* mice, *Muc20* NC and OE NRK-52E cell as well as NC and OE HK2 cell line before and after ML-099 treatment for 24h by ELISA assay. **I** Summarize of possible cascade about how cGAS-STING signaling cascade is influenced by MUC20 through MET and RAS.

To understand how STING is activated in *Muc20* deficient cells, we measured 2′3′ cyclic GMP-AMP (ER) levels, the direct activator of STING synthesized by cGAS from ATP and GTP^47^. ELISA analysis showed significantly higher 2'3'-cGAMP levels in *Muc20* KO cells compared to WT controls **(Figure 8A)**, consistent with increased STING activation. Given that cGAS activity depends on ATP and GTP availability, we hypothesized that GTP levels regulate 2'3'-cGAMP synthesis. Based on previous studies showing that MUC20 interacts with MET and activates RAS signaling^16^, ^48^, ^49^, and considering that RAS cycles between GTP-bound active and GDP-bound inactive states^50^, we proposed a novel mechanism: GTP-bound RAS may sequester cellular GTP, limiting substrate availability for cGAS **(Figure 8B)**. GST-RBD pull-down assays revealed reduced RAS-GTP levels in *Muc20* KO PTECs, suggesting increased free GTP availability for cGAS **(Figure 8C)**. Western blot analysis confirmed decreased MET phosphorylation and reduced expression of downstream effectors MEK1/2 in KO cells, along with lower total MET protein levels **(Figure 8D)**. Overexpression studies in NRK-52E cells validated MUC20's positive regulation of MET pathway activation **(Figure 8D)**. Immunofluorescence demonstrated diminished MET signal intensity colocalized with MUC20 in KO PTECs** (Figure 8E)**, and co-immunoprecipitation confirmed weakened physical interaction between MET and MUC20 in KO lysates **(Figure 8F)**. These results reveal that MUC20 functions as a key regulator of GTP homeostasis through its interaction with MET, influencing innate immune signaling in renal epithelial cells.

To further determine whether Ras activation regulates intracellular 2′3′-cGAMP levels through alterations in the GTP-GDP pool balance, cells were treated with the Ras-related GTPase agonist ML-099, followed by quantitative assessment of intracellular 2′3′-cGAMP by ELISA assay before and after intervention. Meanwhile, we generated a stable MUC20-overexpressing HK2 cell line and compared basal 2′3′-cGAMP levels among the three indicated cell models under unstimulated conditions.

Consistent with our previous observations, basal intracellular 2′3′-cGAMP levels were significantly lower in MUC20-overexpressing cells **(Figure S6A)**. Upon ML-099 treatment for 24 hours, intracellular 2′3′-cGAMP levels were uniformly reduced across all three cell lines, resulting in comparable cGAMP abundance irrespective of MUC20 expression status **(Figure 8H)**. These results indicate that MUC20 suppresses intracellular 2′3′-cGAMP accumulation by promoting Ras activation and stabilization of the Ras-GTP-bound state, thereby reducing the availability of free GTP required for 2′3′-cGAMP synthesis.

Based on prior evidence linking STING activation to mitochondrial oxidative stress^51^, we assessed mitochondrial reactive oxygen species (mtROS) levels in *Muc20*-deficient and *Muc20*-overexpressing cells using MitoSOX staining followed by confocal microscopy and flow cytometric analysis. Upon cisplatin treatment, NRK-52E cells overexpressing *Muc20* exhibited a significant reduction in MitoSOX mean fluorescence intensity compared with control cells. In contrast, genetic ablation of MUC20 resulted in a marked increase in mitochondrial ROS levels. These findings indicate that MUC20 negatively regulates mitochondrial ROS production under stress conditions, thereby providing mechanistic support for its role in restraining STING activation.

Then we further assess the physiological relevance of RAS modulation in kidney fibrosis, we treated UUO mice with ML-099 **(Figure S6B)**. ML-099 significantly attenuated fibrotic lesions without affecting normal kidney structure **(Figure S6C-D)**. Transcriptional analysis also showed downregulation of fibrosis markers *Col1a1* and *Col3a1* **(Figure 8G, Figure S6E)**, confirming the therapeutic potential of RAS activation in renal fibrosis.

In conclusion, our data define a MUC20-MET-RAS regulatory axis that maintains GTP balance, thereby limiting cGAS-mediated 2'3'-cGAMP production and suppressing STING-dependent inflammation **(Figure 8I)**.

### Overexpression of *Muc20* attenuates FA-induced renal fibrosis

**Figure 9:**
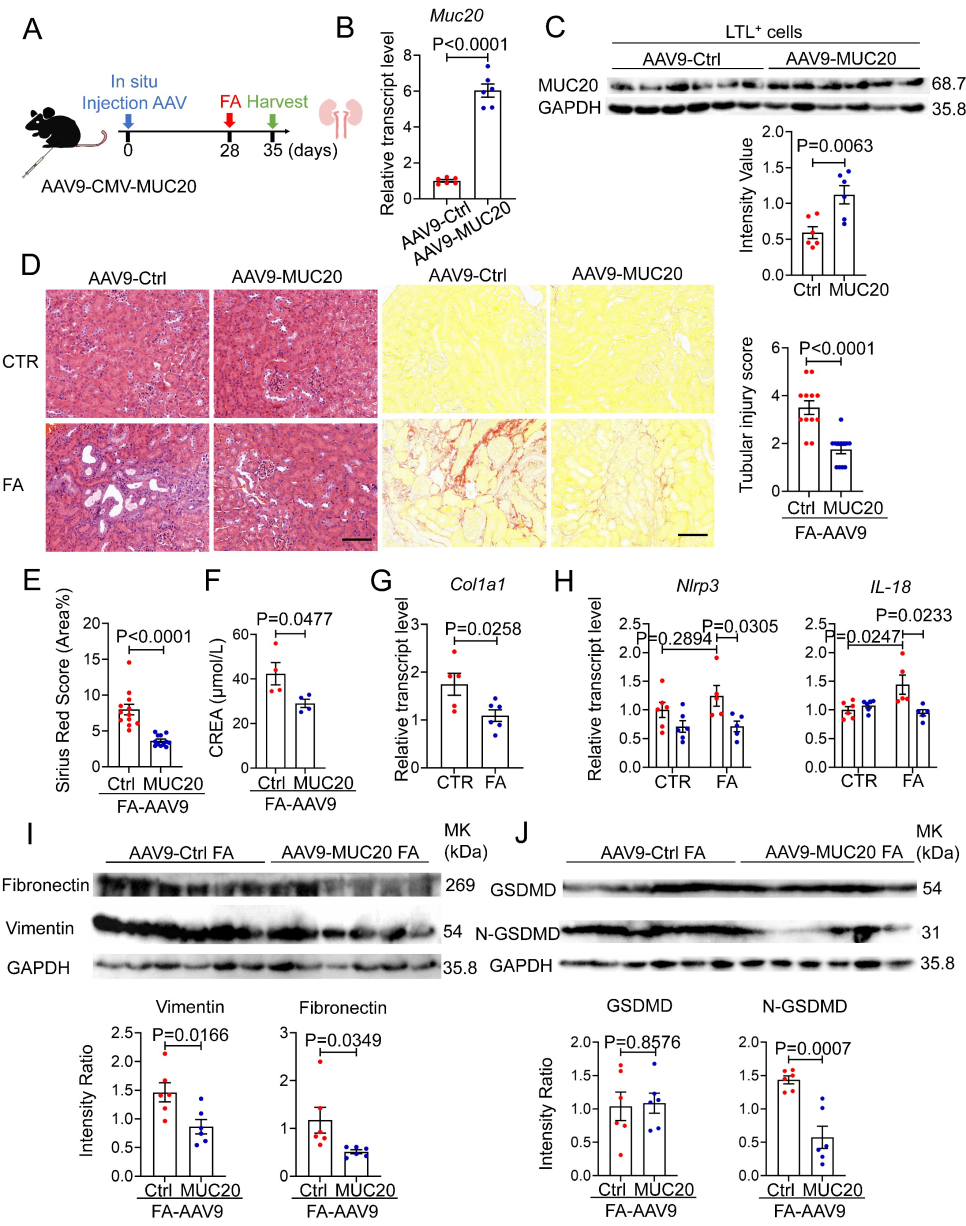
** FA-induced kidney fibrosis was significantly mitigated by *Muc20* overexpression. A** Schematic diagram of *Muc20* overexpression in mice. Eight-week-old male mice were injected with two-type-AAV9 *in situ*. The mice were challenged with FA treatment 4 weeks subsequent to injection. **B** The relative expression levels of *Muc20* in kidneys with two AAV9 injection groups. **C** Western blots of Muc20 in LTL-positive cells from kidneys. (AAV9-Ctrl: n = 6; AAV9-Muc20: n = 6) **D** Left: Kidney sections treated with H&E and Sirus red-staining from two types of AAV9 injected mice after FA treatment. Scale bars: 100 μm. Right: Scoring of renal tubular damage assessed by pathological change according to random HE-stained kidney cortical region sections. **E** Sirius Red staining assessed by percentage of red-stained collagen area in each selected random cortical region field. **F** Serum creatine (CREA) measurement of AAV9-Ctrl and AAV9-Muc20 mice following FA intraperitoneal injection. Samples with hemolysis was excluded. Sham-treated group: AAV9-Ctrl (n = 4) AAV9-Muc20 (n = 4); **G** Relative renal expression of the fibrotic marker *Col1a1* in FA-treated mice injected with AAV9-Ctrl or AAV9-Muc20. **H** The relative expression levels of pyroptosis related markers Nlrp3 and IL-18 in kidneys of AAV9-Ctrl or AAV9-Muc20 injected mice following FA administration. **I** Western blot analysis of fibrosis markers *Fibronectin* and *Vimentin* in kidneys from FA-treated mice injected with AAV9-Ctrl (n = 6) or AAV9-Muc20 (n = 6) **J** Western blots of pyroptosis related markers GSDMD and N-GSDMD in kidneys of AAV9-Ctrl (n = 6) or AAV9-Muc20 (n = 6) injected mice.

To complement our *in vivo* loss-of-function studies, we adopted a gain-of-function approach by injecting adeno-associated virus serotype 9 expressing Muc20 (AAV9-Muc20) or a control vector (AAV9-Ctrl) into the renal cortex of 8-week-old male mice, as previously described^52^, following FA administered to induce kidney injury subsequently **(Figure 9A)**. Four weeks after viral delivery, Muc20 mRNA levels were markedly elevated in renal tissues from AAV9-Muc20-treated mice **(Figure 9B)**. To assess cell-type-specific expression, Lotus tetragonolobus lectin-positive (LTL⁺) proximal tubular epithelial cells were isolated, in which MUC20 protein expression was robustly increased, confirming efficient and targeted transduction of proximal tubules **(Figure 9C)**. Following FA induced kidney injury, mice with tubular MUC20 overexpression exhibited a pronounced reduction in renal fibrosis at the tissue level. *Muc20* overexpression *in vivo* improved tubular dilation, decreased infiltration of inflammatory cells, and attenuated deposition of collagen measured by H&E and Sirius-red staining **(Figure 9D-E)**. qPCR and western blot analyses showed MUC20 overexpression reduced fibrosis extent indicated by *Col1a1*,* Fibronectin*,* Vimentin* transcriptive level **(Figure 9G, Figure 9I).** Consistently, the expression of pyroptosis-associated proteins, including full-length gasdermin D (GSDMD) and its active N-terminal fragment (N-GSDMD), was significantly decreased in *Muc20*-overexpressing kidneys compared with control animals at both transcriptive and protein expression levels **(Figure 9H, Figure 9J)**. Together, these data demonstrate that proximal tubule-restricted overexpression of MUC20 suppresses fibrotic signaling and attenuates pyroptotic cell death *in vivo*, thereby conferring protection against folic acid induced renal injury.

## Discussion

Here, we identify transmembrane mucin MUC20 as a critical protective regulator in kidney injury and fibrosis. Our findings show that *Muc20* expression is significantly downregulated in both human kidney fibrosis samples and mouse models of renal fibrosis. Mechanistically, MUC20 deficiency exacerbates tubular injury by promoting pyroptosis. Within this pathway, MUC20 directly binds MET to sustain RAS activation (RAS-GTP), thereby limiting cytoplasmic GTP availability for cGAS-mediated 2'3'-cGAMP production. This interaction suppresses STING-dependent lysosomal permeabilization and NLRP3 inflammasome activation by maintaining potassium homeostasis. As a result, pharmacological inhibition of NLRP3 or activation of RAS effectively attenuated fibrosis progression in *Muc20* KO mice.

MUC20 fills an urgent need for new therapeutic targets in end-stage renal disease. Although *SOX17*, *UMOD*, *DAB2*, *DACH1* and *MANBA* have been linked to CKD progression^53^, ^54^, the role of transmembrane mucins remains underexplored. Our work uniquely identifies MUC20 as a guardian of tubular integrity through MET/RAS signaling, revealing a novel protective mechanism against fibrosis.

Cell death is a central driver of renal injury and fibrogenesis. Pyroptosis, in particular, drives inflammation through immune cells infiltration and cytokine release^44^, ^55^. It is characterized by cell swelling, membrane rupture^56^, and release of interleukin-1β and IL-18^57^, and contributes to diseases such as obstructive nephropathy and diabetic kidney injury^43^, ^58^-^61^. We found that *Muc20* KO mice exhibit elevated pyroptotic markers (NLRP3, IL-1β, IL-18) in UUO and cisplatin-induced models, linked mechanistically to potassium efflux-driven NLRP3 activation^40^, ^62^. Cytosolic DNA also activates cGAS-STING signaling during sterile inflammation^63^, ^64^, which triggers lysosomal membrane permeabilization, potassium efflux, and pyroptosis^65^, ^66^. Our data extend this model to renal epithelial cells, showing that MUC20 deficiency exacerbates cGAS-STING signaling, leading to lysosomal cell death, potassium loss, and NLRP3 inflammasome assembly.

Although MUC20 is known to regulate tumor immunity^67^, its renal function involves direct MET interaction and downstream signaling^15^. We show that MUC20 loss reduces MET and RAS-GTP levels, increasing free GTP for cGAS to produce 2'3'-cGAMP, which hyperactivates STING and drives pyroptosis. The role of RAS in fibrosis remains controversial. Although RAS proteins regulate proliferation and apoptosis in cancer^68^, they also promote renal fibrosis progression^69^. Studies show oncogenic RAS suppresses ECM genes like fibronectin and collagens^70^, and RAS deletion increases ECM accumulation^71^. These findings suggest TGF-β counteracts RAS effects, consistent with its tumor-suppressive role^70^.

In our study, although MUC20 overexpression significantly modulated downstream inflammatory and pyroptotic responses, we did not observe a consistent change in canonical markers of ER stress in* Muc20* KO kidneys compared with controls. Notably, recent evidence has implicated the STING (stimulator of interferon genes) pathway in the integration of multiple stress signals in kidney injury and fibrosis, including ER stress and mitochondrial dysfunction. For example, in models of acute tubular injury, STING activation has been linked to ER stress and the production of mitochondrial reactive oxygen species, eventually driving NLRP3 inflammasome activation and pyroptotic cell death in tubular cells via a STING/ER stress/mtROS axis^45^. However, the precise relationship between ER stress and chronic fibrotic remodeling in the kidney remains complex. In unbiased transcriptomic analyses of chronic kidney disease models, ER stress pathways, particularly the PERK arm of the unfolded protein response, were associated with fibrotic gene expression, and STING has been identified as an upstream regulator of ER stress in tubule cells during fibrogenesis^72^. These observations suggest that, although ER stress may contribute to fibrotic progression, its activation may not be a dominant mechanism in every context of STING modulation or in response to MUC20 overexpression in our model. Meanwhile, our findings further indicate that loss of MUC20 influences the extent of mitochondrial oxidative stress, potentially acting as an auxiliary pathway through which MUC20 modulates STING signaling and regulates pyroptosis in the process of kidney fibrosis. As a transmembrane mucin primarily localized to the plasma membrane, the precise mechanisms by which MUC20 impacts mitochondrial function remain unclear and warrant further investigation. Future studies dissecting the relative contributions of mitochondrial versus ER stress-dependent STING activation in fibrogenesis will also be crucial for refining targeted therapeutic strategies.

In our studies, MCC950 and ML-099 were employed to interrogate NLRP3 inflammasome activation and RAS-GTP signaling, respectively. We acknowledge that both agents may exert context-dependent off-target effects. MCC950 has been reported to modulate inflammasome-independent pathways under certain conditions^73^, while ML-099 may influence additional small GTPase-dependent processes as a Ras-related GTPase activator. Therefore, we selected these pharmacological tools as complementary mechanistic probes rather than definitive pathway-specific inhibitors. Importantly, the consistency between pharmacological modulation and genetic manipulation of MUC20 across multiple *in vitro* and *in vivo* systems supports our conclusions. Future studies employing cell type-specific genetic models will be required to further refine pathway specificity.

In summary, our multidisciplinary approach-combining clinical samples, genetically engineered mice, and molecular profiling-identifies MUC20 as a key regulator of renal cytoprotection and a promising therapeutic target. Modulating the MUC20 effector axis, via RAS activation (e.g., ML-099) or pyroptosis inhibitor (e.g., MCC950), offers potential strategies to slow CKD progression, particularly in patients with MUC20 loss-of-function variants. Our discovery of mucin-mediated immunomodulation via the MET-RAS-cGAS-STING-pyroptosis axis provides a novel framework for targeting inflammation in kidney disease and other chronic inflammatory conditions.

## Methods

### Mouse kidney RNA-seq data

Total RNA was extracted from mouse kidney tissues using TRIzol reagent (Thermo Fisher Scientific, #15596018) following instructions. RNA integrity and purity were assessed using an Agilent 2100 Bioanalyzer with RNA 6000 Nano LabChip kits (Agilent, #5067-1511). Samples with an RNA integrity number (RIN) greater than 7.0 were used for subsequent library construction. Poly(A)+ RNA was isolated using Dynabeads Oligo(dT) magnetic beads (Thermo Fisher Scientific) and fragmented by magnesium-mediated hydrolysis (NEB Magnesium RNA Fragmentation Module, #E6150) at 94 °C for 5-7 min. First-strand cDNA synthesis was performed using SuperScript II reverse transcriptase (Invitrogen). Sequencing libraries were prepared according to standard protocols, quantified by qPCR, and subjected to paired-end sequencing (2 × 150 bp) on an Illumina NovaSeq 6000 platform (LC-Bio Technology, Hangzhou, China), yielding millions of paired-end reads per sample. Raw reads were quality-filtered and aligned to the mouse reference genome using HISAT2 (v2.2.1) with default parameters. Library preparation, sequencing, and primary bioinformatic analyses were conducted by LC-Bio Technology.

### Animal models

Male C57BL/6 mice aged either 2-4 weeks (for primary tubular epithelial cell isolation) or 8-10 weeks (for in vivo experiments) were housed under specific pathogen-free. All animal procedures were approved by the Institutional Animal Care and Use Committee of Fudan University (Protocol #2024306) and conducted according to NIH guidelines. Muc20 knockout mice were produced via CRISPR/Cas9-mediated genome editing by co-injection of Cas9 mRNA and sgRNAs (East China Normal University). Supplementary Fig. 2 and Supplementary Table 1 contain sgRNA sequences and genotyping primers we used in this research.

For the unilateral ureteral obstruction (UUO) model, WT and *Muc20⁻/⁻* mice were treated with left ureter ligation at 8 weeks after birth, with sham-operated mice serving as controls. Kidneys were harvested on postoperative days 4 or 7. For folic acid and cisplatin-induced nephropathy, mice received a single intraperitoneal injection of folic acid (250 mg/kg, dissolved in 300 mM sodium bicarbonate), cisplatin (7mg/kg, dissolved in normal saline) and were euthanized 7 days later in FA mice models and 28 days later in cisplatin models. Cisplatin-induced acute nephrotoxicity was established by a single intraperitoneal injection of cisplatin (25 mg/kg), with tissue collection at day 3 post-injection.

For pharmacological studies, WT and *Muc20⁻/⁻* mice received intraperitoneal injections of MCC950 or the Ras-related GTPase activator ML099 (20 mg/kg; stock solution 7 mM in DMSO) three times per week (every 48 days). Treatments were initiated prior to UUO surgery and continued until tissue harvest.

### AAV9-mediated renal overexpression of *Muc20*

For renal-specific overexpression, 8-week-old male C57BL/6J mice were anesthetized with isoflurane and injected with AAV9-CMV-MUC20 or control AAV9 (2.5 × 10¹³ vg/mL) into five distinct sites within the renal cortex (5 μL per site) using a glass micropipette.

### Serum biochemistry

Peripheral blood was collected, allowed to clot at room temperature for 45 min, and centrifuged at 6,000 rpm for 15 min at 4 °C. Serum creatinine and blood urea nitrogen (BUN) levels were measured using automated chemistry analyzers (Chemray 240, Leidu Biotechnology; Epoch Microplate Spectrophotometer, BioTek) according to manufacturer protocols. All measurements were performed by Servicebio Biotechnology (Wuhan, China).

### Histopathology analysis

Kidneys were harvested, rinsed in ice-cold PBS, and were fixed in 10% neutral buffered formalin, paraffin-embedded, sectioned at 4 μm, and stained with hematoxylin and eosin (H&E) or Picrosirius Red (Solarbio, #G1472). Tubular injury was evaluated using a semi-quantitative scoring system based on eight pathological parameters, as described previously with minor modifications in a blinded manner^74^. Two independent pathologists performed all assessments.

### Quantitative real-time PCR

Total RNA from tissues or cultured cells was extracted accompanied with TRIzol reagent. cDNA was synthesized from 1 μg RNA using HiScript III RT SuperMix (Vazyme). Quantitative PCR was performed using SYBR Green Master Mix. Gene expression was normalized to GAPDH. Primer sequences are listed in Supplementary Table 2.

### Lentivirus production and stable cell line generation

Lentiviruses were produced in HEK293T cells via PEI-mediated co-transfection of transfer plasmid (52961-MUC20) with packaging plasmids pMD2.G and psPAX2. Viral supernatants were collected, filtered, concentrated by ultracentrifugation, and stored at -80 °C. NRK-52E cells were transduced and selected with puromycin (5 μg/mL) for 14 days. Stable clones were validated by immunoblotting.

### Cell culture and treatments

NRK-52E cells were cultured in DMEM supplemented with 5% FBS. Primary renal tubular epithelial cells were isolated from 2-4-week-old mice by collagenase digestion and maintained in RPMI 1640 supplemented with 10% FBS, EGF, 1 × ITS, and antibiotics. All cell lines were routinely tested and confirmed to be mycoplasma-free. For cytotoxicity assays, cells were treated with nigericin (5 μM) for 24 h or cisplatin (20 μM) for 16 h.

### Western blotting and immunofluorescence

Protein lysates were prepared in RIPA buffer supplemented with protease and phosphatase inhibitors. Equal amounts (20 μg/lane) of protein were resolved by SDS-PAGE, transferred to PVDF membranes (0.25 μm pore size), and probed with indicated antibodies provided in supplementary table 3. For immunofluorescence, cells were fixed, permeabilized, blocked, and incubated with primary and Alexa Fluor-conjugated secondary antibodies. Nuclei were counterstained with DAPI.

### Cell viability and cytotoxicity assessment

Cell viability was assessed using Trypan Blue exclusion (Thermo Fisher, #T10282), LDH release (Beyotime, #C0016), and CCK-8 assays. Apoptotic cells were detected by TUNEL staining (Beyotime, #C1090). Annexin V/SYTOX staining was used to distinguish cell death modalities. Intracellular potassium flux was measured using PBFI-AM (Maokangbio #MX4510). Ras activation was analyzed using a Ras-GTP pull-down assay (NewEast Biosciences #81101).

### Statistical analysis

Data are presented as mean ± SEM from at least three independent experiments. Statistical analyses were performed using GraphPad Prism 9.0. Differences were assessed using two-tailed Student's t-tests or one- or two-way ANOVA with Tukey's post hoc test. P < 0.05 was considered statistically significant.

### Study approval

Rat and mice were raised and maintained in a barrier facility. Animal studies were conducted under approved by the Institutional Animal Care and Use Committee of the Fudan University and conducted in accordance with ARRIVE guidelines.

## Supplementary Material

Supplementary figures and tables.
