# Supplementary material for: MUC20 alleviates kidney fibrosis by modulating pyroptosis through the MET/RAS/STING axis
Source: Theranostics. 2026 Feb 4;16(8):4374–93. doi: 10.7150/thno.123986 (PMC12906183; doi:10.7150/thno.123986)
Supplement: Supplementary file 1 — Supplementary figures and tables. [file thnov16p4374s1.pdf]

Supplementary Materials for

MUC20 alleviates kidney fibrosis by modulating pyroptosis through the MET/RAS/STING axis

Jiaxin Huang[1,#], Zhoutong Chen[1,#], Fengbo Zhong[2,#], Rui Zheng[1], Dexin Zhang[1], Jingyi Su[3], Yi Zhong[2], Xiaoliang Fang[1], Dali Li[2], Yuting Guan[2,*], Hongquan Geng[1,*]

[*]**Correspondence**

Hongquan Geng, MD, PhD

Department of Urology, Children's Hospital of Fudan University, Shanghai, 201102, China

E-mail: genghongquan@fudan.edu.cn

Yuting Guan, PhD

Shanghai Frontiers Science Center of Genome Editing and Cell Therapy, Shanghai Key Laboratory of Regulatory Biology, Institute of Biomedical Sciences and School of Life Sciences, East China Normal University, Shanghai, 200241, China

E-mail: ytguan@bio.ecnu.edu.cn

This PDF file includes:

Figs. S1 to S7

Tables S1 to S2

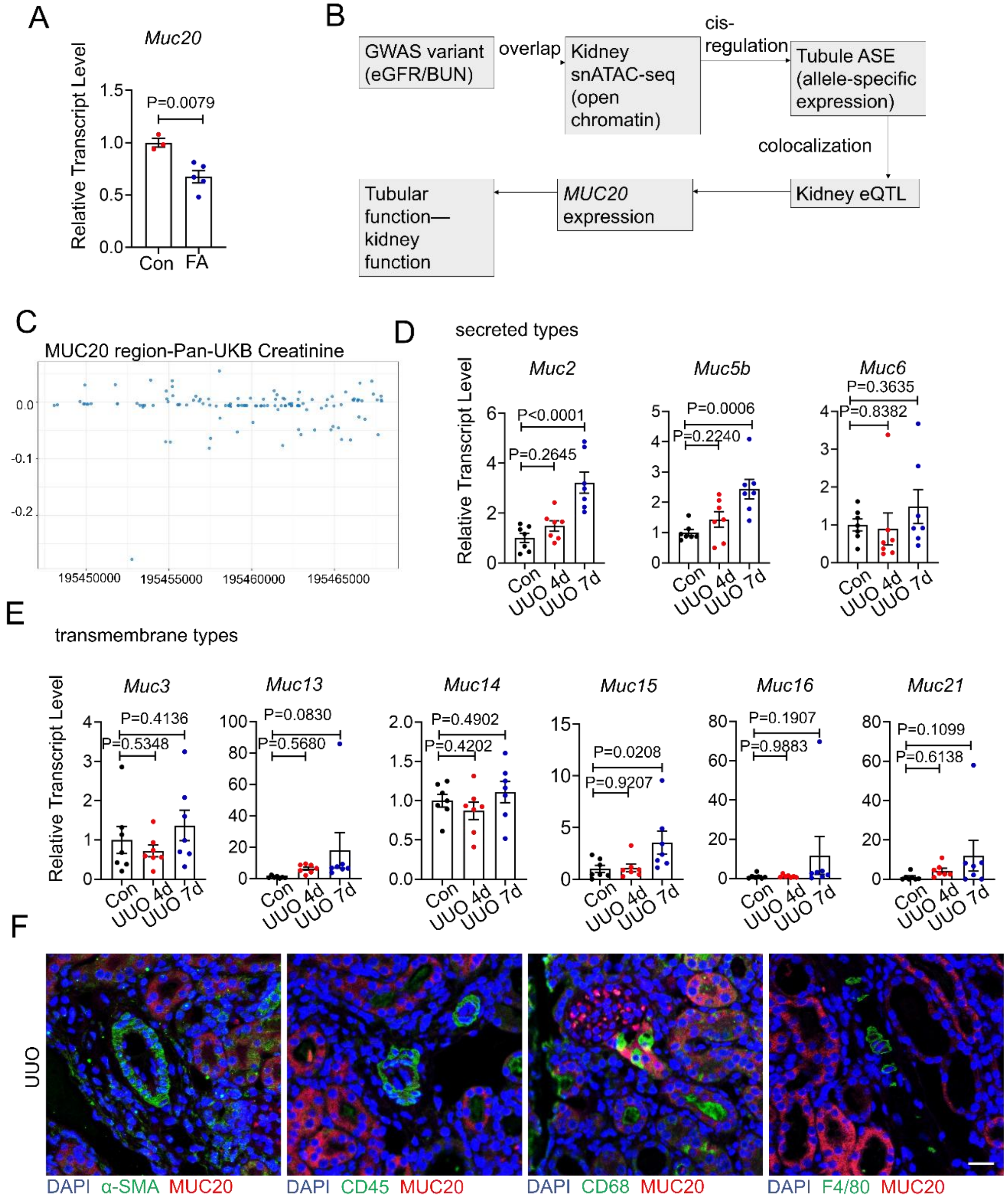

Supplementary figure 1. Other mucin family members detection in UUO induced kidney fibrosis models

a Relative transcript level of fibrosis markers *Muc20* in kidneys of 7 days after folic acid intraperitoneal injection treated wild mice with vehicle injection treated as control. Sham-treated group: WT (n=3), FA treated group: WT (n=5).

b Integrative causal framework linking MUC20 to kidney function.
c Genetic association plot for the MUC20 gene region with serum creatinine levels from the Pan-UK Biobank (Pan-UKB) project. The Y axis represents the estimated effect of each genetic variant on serum creatinine levels.
d Secreted mucin family members including *Muc2*, *Muc5b, Muc6* transcriptive level in kidneys of UUO-4-day, UUO-7-day surgery treated wild type and sham-treated as control. Sham-treated group: WT(n=7); UUO-4-day treated group: WT (n=7); UUO-7-day treated group: WT(n=7).
e Relative transcript level of transmembrane typed mucin family members including *Muc3*, *Muc13*, *Muc14*, *Muc15*, *Muc16*, *Muc21* in kidneys of UUO-4-day, UUO-7-day surgery treated wild type and sham-treated as control. Sham-treated group: WT(n=7); UUO-4-day treated group: WT (n=7); UUO-7-day treated group: WT(n=7).
f Immunofluorescent double staining of MUC20 and fibroblast cell, immune cell and macrophage markers α-SMA, CD45, CD68 and F4/80 for co-location detection in UUO-4day-challenged wild type mouse kidney sections. Scale bar: 25 μm.

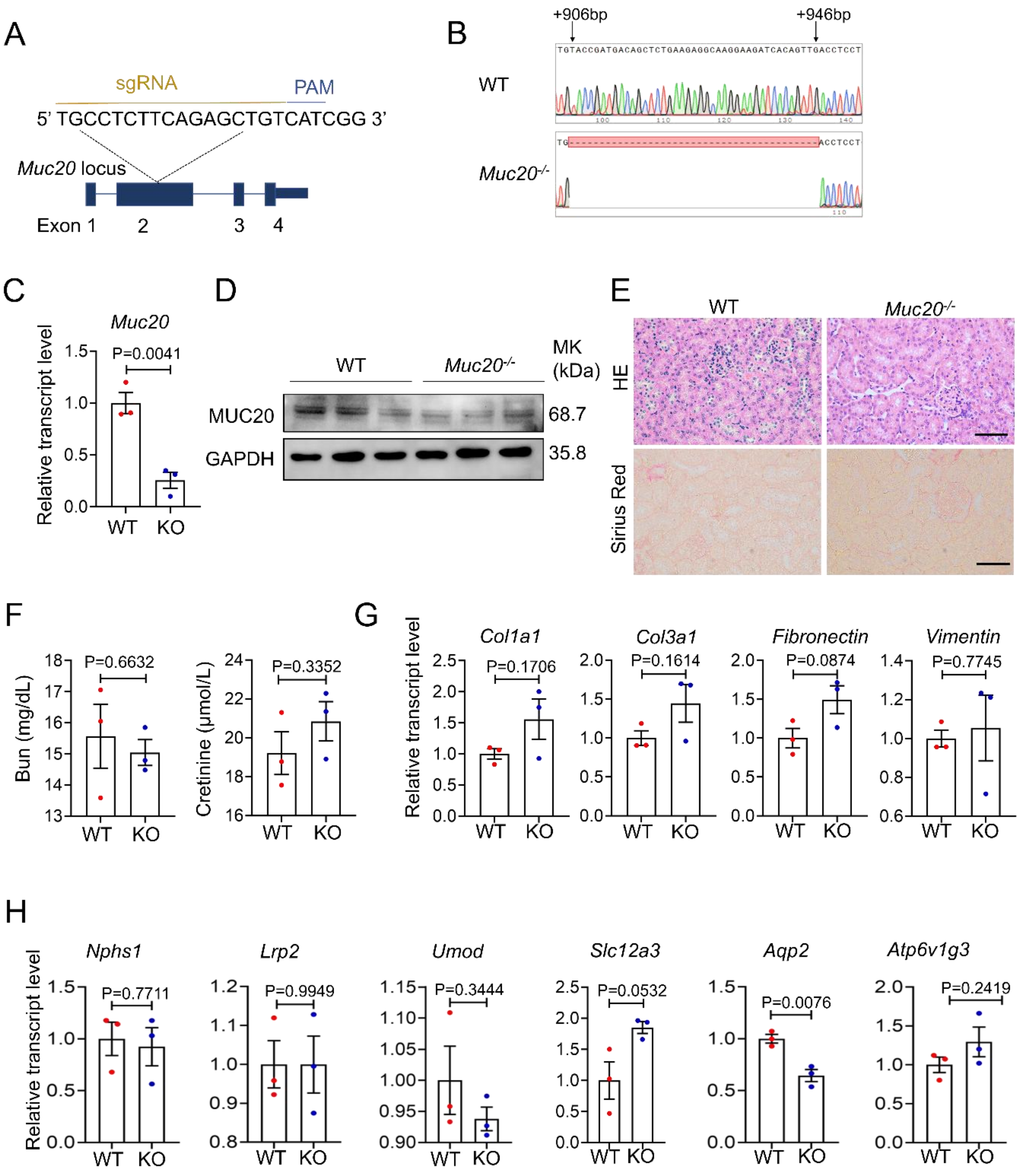

Supplementary figure 2. Basal line of CRISPR-Cas9 edited *Muc20* knockout mice.

a Sequence of donor sgRNA compared with WT and the *Muc20* exon map of the selected donor. **b** Sanger sequencing TIDE focused on the deleted region of MUC20 in kidneys acquired from control wide type and CRISPR-Cas9 based edited *Muc20* KO mice at basal line without any treatment.

c Relative transcript level of *Muc20* in kidneys of wild type and *Muc20*$^{-/-}$ mice without treatment at basal line. Sham-treated group: WT(n=3), *Muc20*$^{-/-}$ (n=3).

d Western blots of MUC20 in kidneys acquired from control and CRISPR-Cas9 edited mice at basal line without any treatment. Blots were compared with GAPDH as conference.

e Representative images of HE-stained and Sirius Red-stained kidney sections from control and *Muc20*$^{-/-}$ mice without any treatment at basal line. Scale bar: 100μm.

f Serum blood urea nitrogen (BUN) and serum creatinine measurement of wild type and *Muc20*$^{-/-}$ mice without treatment at basal line.

g Fibrosis indicators *Col1a1, Col3a1, Fibronectin* and *Vimentin* transcriptive leve; in kidneys of wild type and *Muc20*$^{-/-}$ mice without treatment. Sham-treated group: WT(n=3), *Muc20*$^{-/-}$ (n=3).

h Relative transcript level of kidney development related markers including *Nphs1*, Lrp2, *Umod*, *Slc12a3*, *Aqp2*, *Atp6v1g3* in kidneys of wild type and *Muc20*$^{-/-}$ mice without treatment. Sham-treated group: WT(n=3), *Muc20*$^{-/-}$ (n=3).

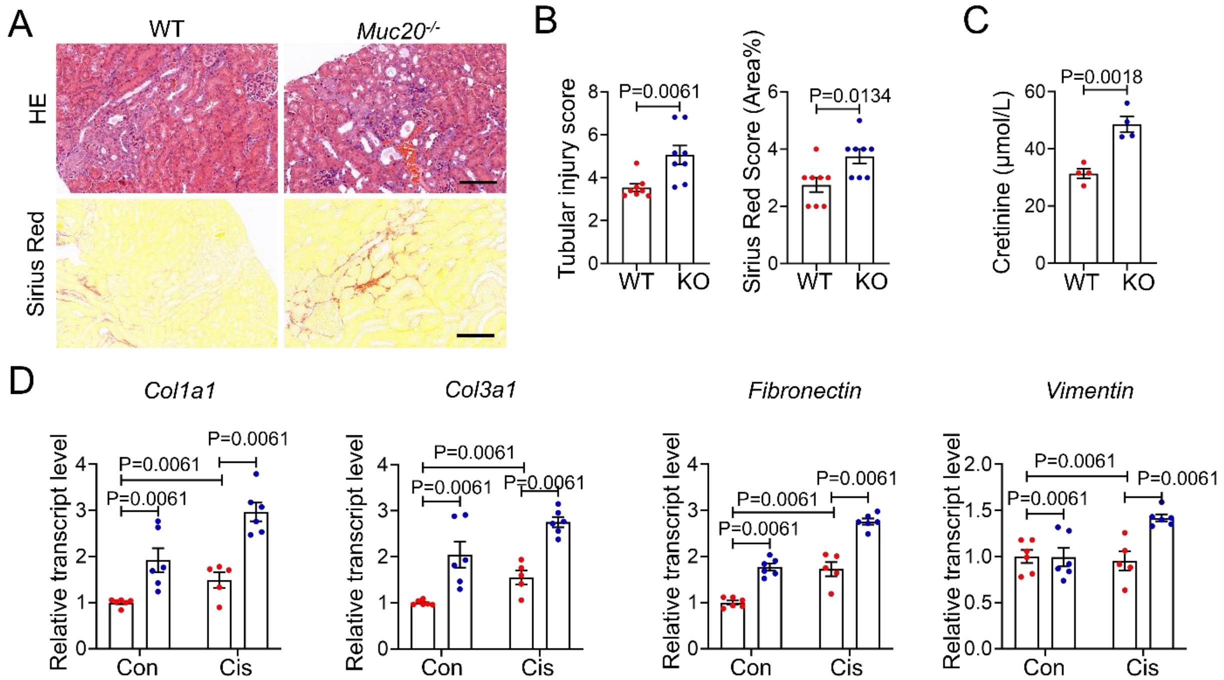

Supplementary figure 3. Deficiency in *MUC20* exacerbated renal injury in iconic chronic kidney injury mice induced by cisplatin at low dose for a month.

a Representative images of HE-stained and Sirius Red-stained kidney sections from WT as control and *Muc20*$^{-/-}$ mice following cisplatin treatment at 7mg/kg for 4 weeks (single dose per week). Sham-treated group: WT(n=6), *Muc20*$^{-/-}$ (n=6); Cisplatin treated group: WT (n=5), *Muc20*$^{-/-}$ (n=6). Scale bar: 100 μm.

b Left: scoring of renal tubular damage assessed by pathological features according to random HE-stained kidney cortical region sections. Right: scoring of Sirius Red staining assessed by percentage of red-stained collagen area in each selected random cortical region field.

c Serum creatine (CREA) measurement of wild type and *Muc20*$^{-/-}$ mice following cisplatin peritoneal injection at 7mg/kg for 4 weeks (single dose per week). Samples with hemolysis was excluded. Sham-treated group: WT(n=6), *Muc20*$^{-/-}$ (n=6); Cisplatin treated group: WT (n=5), *Muc20*$^{-/-}$ (n=6).

d Fibrosis markers *Col1a1, Col3a1, Fibronectin* and *Vimentin* expression in kidneys of wild type and *Muc20*$^{-/-}$ mice with or without treatment of cisplatin intraperitoneal injection at 7mg/kg for 4 weeks (one dose per week). Sham-treated group: WT(n=6), *Muc20*$^{-/-}$ (n=6); Cisplatin treated group: WT (n=5), *Muc20*$^{-/-}$ (n=6).

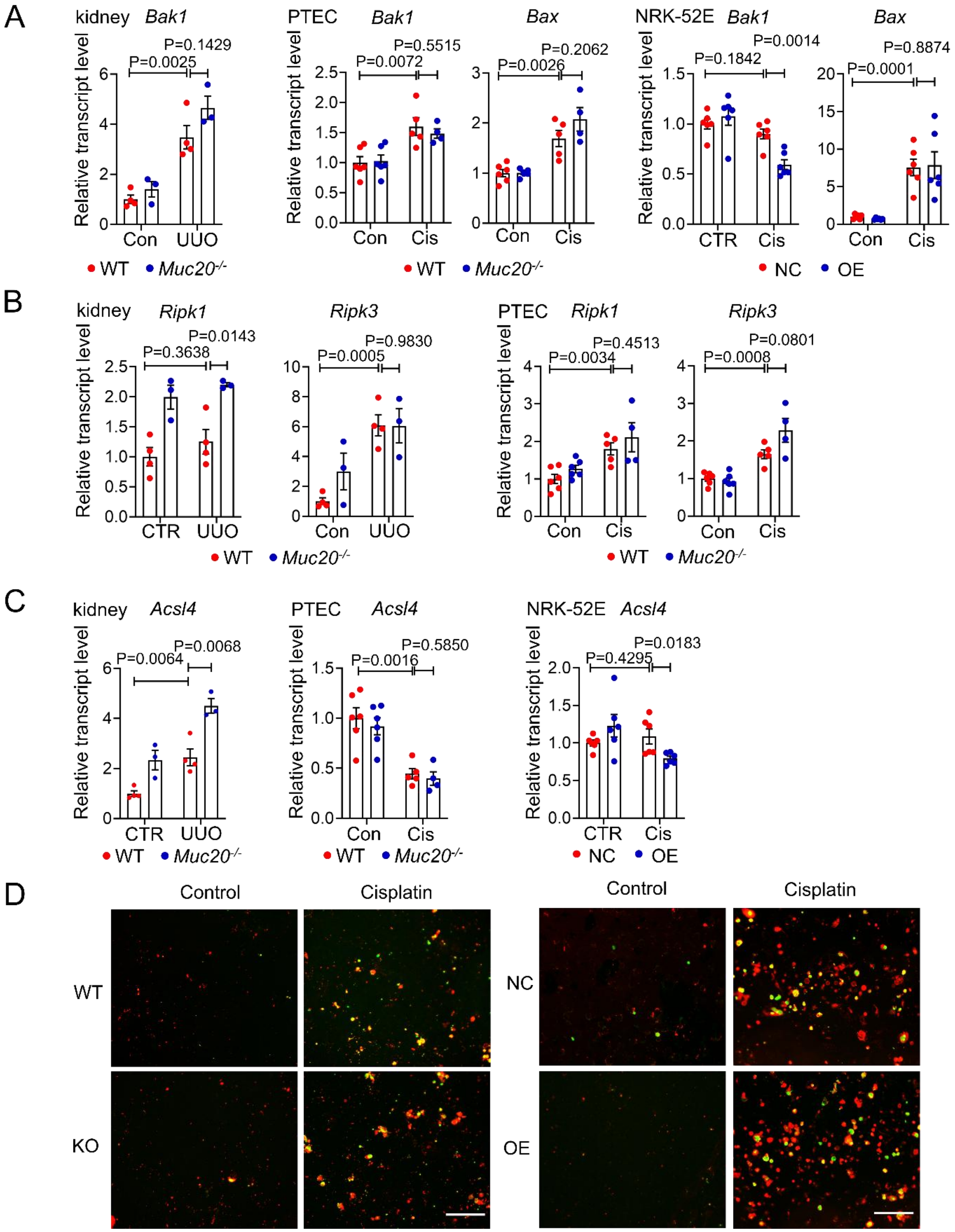

Supplementary Figure 4. Deficiency in *MUC20* not interfere process of cell apoptosis, necrosis or ferroptosis

a Relative transcript level of apoptosis related markers including *Bak1*, *Bax* in kidneys of UUO-4-day surgery treated wild type and *Muc20*$^{-/-}$ mice with sham-treated as control; in primary tubular epithelial cell acquired from 3-week-old WT and *Muc20*$^{-/-}$ mice following sham treatment or Cisplatin treatment; and in NC cell and OE cell following sham treatment or Cisplatin treatment. Kidney tissues: Sham-treated group: WT (n=4), *Muc20*$^{-/-}$ (n=3); UUO-4-day treated group: WT (n=4), *Muc20*$^{-/-}$ (n=3). PTEC: Sham-treated group: WT (n=6), *Muc20*$^{-/-}$ (n=6); Cisplatin treatment group: WT (n=5), *Muc20*$^{-/-}$ (n=4). NRK-52E: Sham-treated group: NC (n=6), *OE* (n=6); Cisplatin treatment group: NC (n=6), *OE* (n=6).

b Relative transcript level of necroptosis related markers including *Ripk1*, *Ripk3* in kidneys of UUO-4-day surgery treated wild type and *Muc20*$^{-/-}$ mice with sham-treated as control; in primary tubular epithelial cell acquired from 3-week-old WT and *Muc20*$^{-/-}$ mice following sham treatment or Cisplatin treatment. Kidney tissues: Sham-treated group: WT (n=4), *Muc20*$^{-/-}$ (n=3); UUO-4-day treated group: WT (n=4), *Muc20*$^{-/-}$ (n=3). PTEC: Sham-treated group: WT (n=6), *Muc20*$^{-/-}$ (n=6); Cisplatin treatment group: WT (n=5), *Muc20*$^{-/-}$ (n=4).

c Relative transcript level of ferroptosis related markers *Acsl4* in kidneys of UUO-4-day surgery treated wild type and *Muc20*$^{-/-}$ mice with sham-treated as control; in primary tubular epithelial cell acquired from 3-week-old WT and *Muc20*$^{-/-}$ mice following sham treatment or Cisplatin treatment; and in NC cell and OE cell following sham treatment or Cisplatin treatment. Kidney tissues: Sham-treated group: WT (n=4), *Muc20*$^{-/-}$ (n=3); UUO-4-day treated group: WT (n=4), *Muc20*$^{-/-}$ (n=3). PTEC: Sham-treated group: WT (n=6), *Muc20*$^{-/-}$ (n=6); Cisplatin treatment group: WT (n=5), *Muc20*$^{-/-}$ (n=4). NRK-52E: Sham-treated group: NC (n=6), *OE* (n=6); Cisplatin treatment group: NC (n=6), *OE* (n=6).

d Representative of Annexin V-mCherry SYTOX green cell apoptosis detection assay on NC cell and OE cell following sham treatment or Cisplatin treatment for 16 hours. Scale bar: 100 μm.

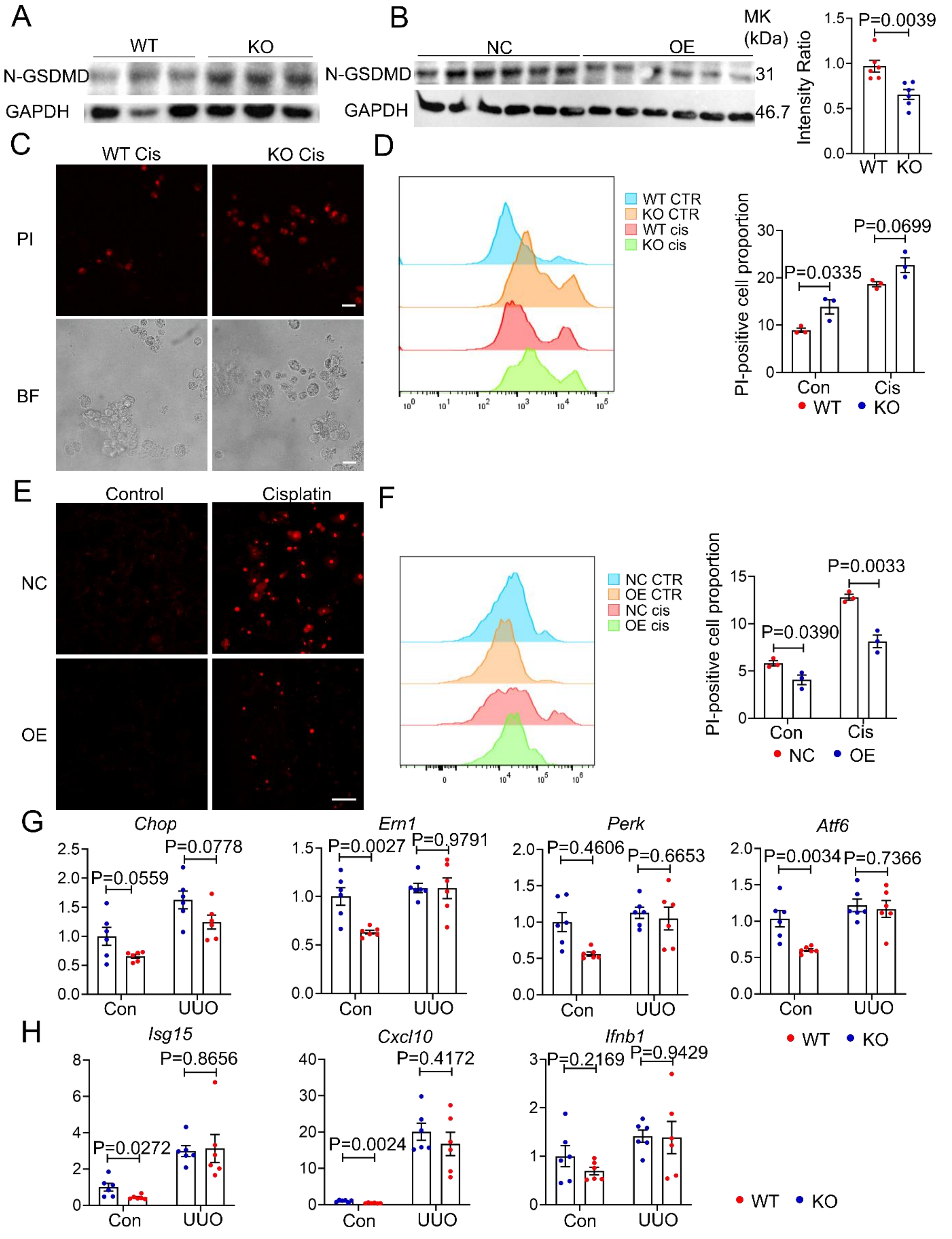

Supplementary Figure 5. MUC20 deficiency specifically potentiates pyroptosis but not ER stress or IFN production.

a Western blots for protein level detection of cleaved N-terminal GSDMD (N-GSDMD) in primary tubular epithelial cell without treatment at basal line acquired and cultured from 3-week-old WT (n=3) and *Muc20*$^{-/-}$ mice (n=3) without treatment at basal line.

b Western blots for protein level detection of cleaved N-terminal GSDMD (N-GSDMD) in MUC20 NC and OE cell without treatment at basal line

c Representative images of Propidium iodide (PI) staining assay for membrane permeability detection on primary tubular epithelial cell following Cisplatin treatment for 16 hours. Scale bar: 100 μm.

d Quantification of PI-positive cell proportion of PI staining, reflecting membrane permeability. Data are from three independent experiments (mean ± SEM). Statistical significance was determined by an unpaired t-test.

e Representative images of Propidium iodide (PI) staining assay for membrane permeability detection on MUC20 NC and OE cell line following sham treatment or Cisplatin treatment for 16 hours. Scale bar: 100 μm.

f Quantification of PI-positive cell proportion of PI staining, reflecting membrane permeability. Data are from three independent experiments (mean ± SEM). Statistical significance was determined by an unpaired t-test.

g Relative transcript level of ER stress related markers including *Chop, Ern1, Perk,* and *Atf6* in kidneys of UUO-4-day surgery treated wild type and *Muc20*$^{-/-}$ mice with sham-treated as control; Sham-treated group: WT (n=6), *Muc20*$^{-/-}$ (n=6); UUO-4-day treated group: WT (n=6), *Muc20*$^{-/-}$ (n=6).

h Relative transcript level of IFN synthesis pathway related markers including *Isg15, Cxcl10, Ifnb1* in kidneys of UUO-4-day surgery treated wild type and *Muc20*$^{-/-}$ mice with sham-treated as control; Sham-treated group: WT (n=6), *Muc20*$^{-/-}$ (n=6); UUO-4-day treated group: WT (n=6), *Muc20*$^{-/-}$ (n=6).

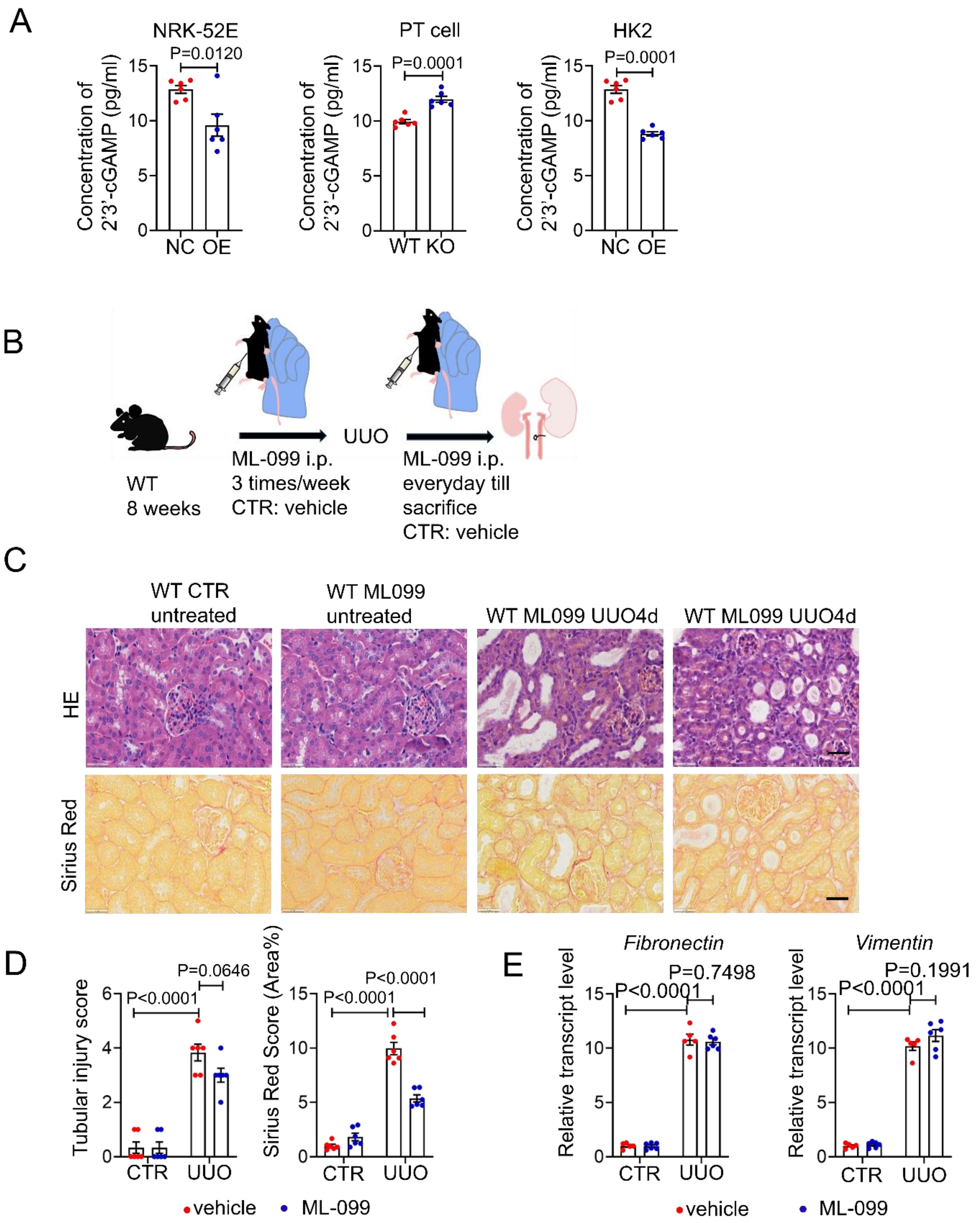

Supplementary figure 6. Ras-related GTPase activator ML-099 could attenuate kidney fibrosis

a Detection of concentration of intracellular 2'3'-cGAMP in primary tubular epithelial cell acquired and cultured from 3-week-old WT and *Muc20*$^{-/-}$ mice, *Muc20* NC and OE NRK-52E cell as well as NC and OE HK2 cell line without treatment at basal line for concentration comparison by ELISA assay.

b Pipeline and illustrators of Ras-related GTPase activator ML-099 injection procedure and construction of kidney fibrosis model.

c HE-stained and Sirius Red-stained kidney sections from WT mice following sham, sham with activator ML099 treatment, UUO-4-day surgery treatment and UUO-4-day surgery with activator ML099 injection. Scale bar: 20 μm.

d Left: Scoring of renal tubular damage assessed by loss of the brush border, tubular dilation, inflammatory cell infiltration degree according to random HE-stained kidney cortical region sections. Right: Scoring of Sirius red staining assessed by area percentage of red-stained fibrin.

e Fibrosis markers *Fibronectin, Vimentin* expression in kidneys of UUO-4-day surgery treated wild type mice with or without activator injection and sham-treated as control. Sham-treated group: vehicle (n=6), ML099 injection (n=6); UUO-4-day treated group: vehicle (n=6), ML099 injection (n=6);

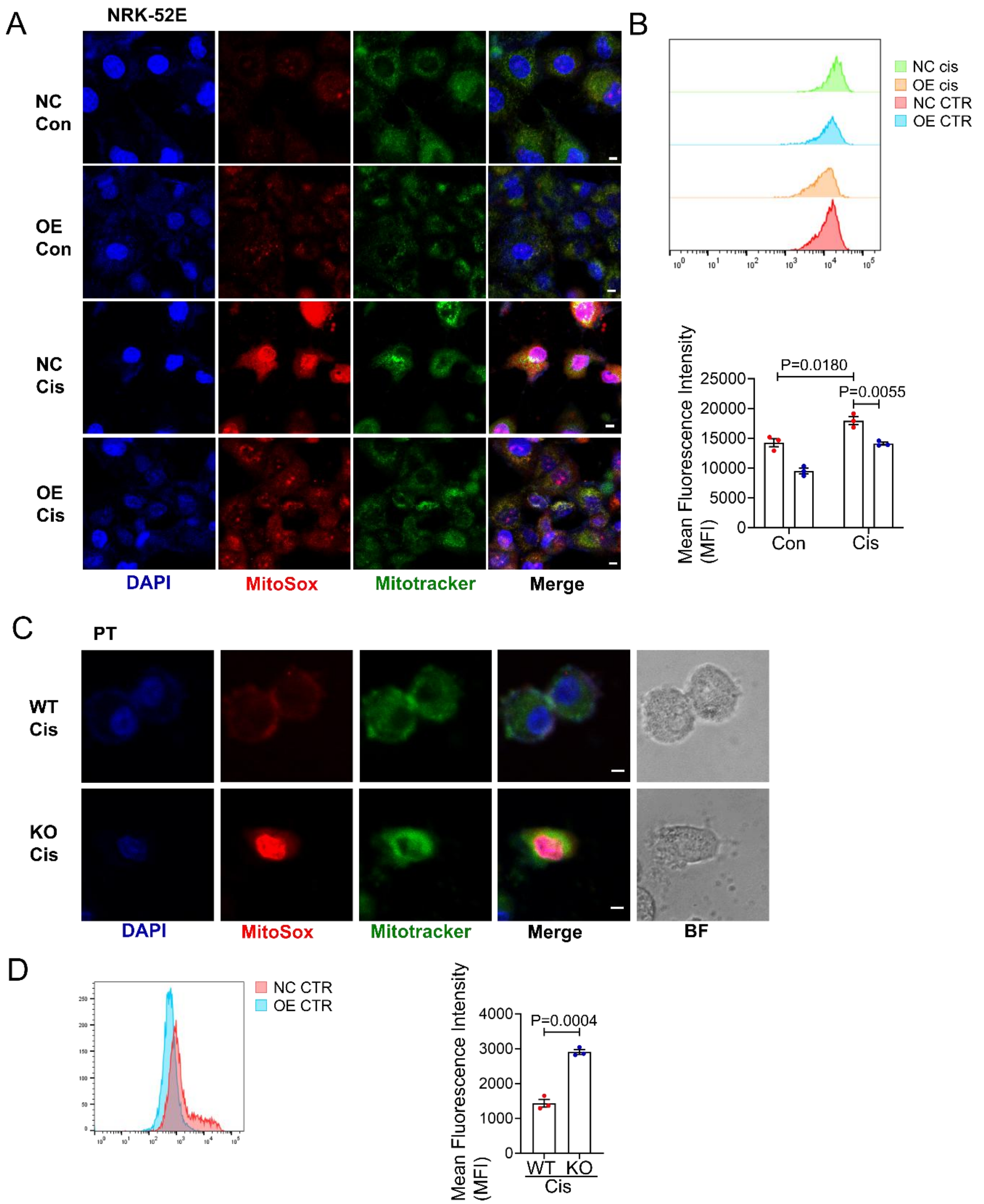

Supplementary Figure 7. MUC20 deficiency promotes mitochondrial reactive oxygen species production

a Representative images of MitoSOX staining, MitoTracker fluorescence within MUC20 NC and OE NRK-52E cells following sham and cisplatin treatment. Scale bar: 50 μm.

b Quantitative analysis of mitochondrial superoxide levels expressed as the median fluorescence intensity (MFI) of MitoSOX Red. Data are pooled from three independent experiments.
c Representative images of MitoSOX staining, MitoTracker fluorescence within WT and KO PT cells following sham and cisplatin treatment. Scale bar: 50 μm.
d Quantitative analysis of mitochondrial superoxide levels expressed as the median fluorescence intensity (MFI) of MitoSOX Red. Data are pooled from three independent experiments.

Table S1. sgRNA and identification primer sequence

**For deletion of *Muc20* gene in mice**	
Slc3a1-sgRNA	TGCCTCTTCAGAGCTGTCATCGG
**For genotyping of *Muc20* KO mice**	
Muc20-F	TTCTGACCTCACGGTTATGC
Muc20-R	CTCGTGAGCTGCTGAGGAC

Table S2. qPCR primer sequences

**Mouse Primers**	
Muc20-F	AAAACCCAAACATCACCTTAACC
Muc20-R	GGTCAGCCGTACAAGGAG
GAPDH-F	ATGCCAAAGTTGTCATGGAT
GAPDH-R	ATGTTTGTGATGGGTGTGAA
Col1a1-F	TGCCTGGACCTCCTGGCGAGCGT
Col1a1-R	AGCAGGTCCGGGAGCACCACGTT
Col3a1-F	ACAGCTGGTGAACCTGGAAG
Col3a1-R	ACCAGGAGATCCATCTCGAC
Fibronectin-F	ACAAGGTTCGGGAAGAGGTT
Fibronectin-R	CCGTGTAAGGGTCAAAGCAT
Vimentin-F	GGAGGCCACGAACTTCACTCT
Vimentin-R	GGGATGCAACACCTATTGTCAGT
Muc1-F	ACATCTTTCCAACCCAGGAC
Muc1-R	GAGACTGCTACTGCCATTACC
Muc3-F	GCAGAAGGGCGATAAGTGGT
Muc3-R	GTGATCAGGCAGAGGCACT
Muc4-F	AATGTTCCTGCCTATACTGCC
Muc4-R	TTGTATGGTTCCTGGGTCAC
Muc13-F	GCAACCCTAACCCCTGTAAAG
Muc13-R	CACTCATGCTAATGTCCCCAG
Muc14-F	CGAAAATCACTGCAACACCC
Muc14-R	CGATAACCACAGGCAAAATG
Muc15-F	CCCAAATACATCAGACACCCC
Muc15-R	GAATCTGTTTTCCGTTGTCCAC
Muc16-F	GCTCTCCTCTGGTGCCTTTC
Muc16-R	TTGAACAAAGACCAAAGCAGTC
Muc18-F	TCAACCCCACTTCACTATCAAC

Muc18-R	ACAGGAGCCCATTTTCATCG
Muc21-F	CCACCCACACTTCCTCTAGC
Muc21-R	AGAGACAGGTATCTTCTCACATAAA
Muc2-F	CGACTGTGAGCAGTGTGTCT
Muc2-R	AAAGGTGGTGATGTGGGAGC
Muc5B-F	CACTGTATGGCTGAGAATGGAG
Muc5B-R	TGCACTTGACAGGTACTTGAG
Muc6-F	ATACTCTTCTCCAGAGCCCACA
Muc6-R	GTCTCTGAGTGCGAGTAACCC
Bak1-F	TGATATTAACCGGCGCTACG
Bak1-R	AGCTGATGCCACTCTTAAATAGG
Bax-F	TTGGAGATGAACTGGACAGC
Bax-R	CAGTTGAAGTTGCCATCAGC
Nlrp3-F	CTCCAACCATTCTCTGACCAG
Nlrp3-R	ACAGATTGAAGTAAGGCCGG
IL1b-F	GCAACTGTTCCTGAACTCAACT
IL-1b-R	ATCTTTTGGGGTCCGTCAACT
Caspase1-F	TCTGTATTCACGCCCTGTTG
Caspase1-R	GATAAATTGCTTCCTCTTTGCCC
Acsl4-F	TTGGCTACTTACCTTTGGCTC
Acsl4-R	AATCACCCTTGCTTCCCTTC
IL-18-F	CGACTTCACTGTACAACCGC
IL-18-R	TGGGGTTCACTGGCACTTTG
Sting-F	GAGAACGGACAGCCAGCAG
Sting-R	CAAGTGTCCGGCAGAAGAGT
**Rat Primers**	
Muc20-F	TGATGCACCAGCTCCGC
Muc20-R	GGCCGTGGTTTGTTGGTAAC
GAPDH-F	CCATCAACGACCCCTTCATT
GAPDH-R	GACCAGCTTCCCATTCTCAG
Nlrp3-F	TCCCTGGGATTTCTCCACAAC
Nlrp3-R	CAGCAGTTCACCAGTCTGGAAG
IL1b-F	TGCAGGCTTCGAGATGAAC
IL-1b-R	GGGATTTTGTCGTTGCTTGTC
Caspase1-F	CACATGAAAGAATATGCCTGGTC
Caspase1-R	GTCCTGGGAAGAGGTAGAAAC
Acsl4-F	TTGGCTACTTACCTTTGGCTC
Acsl4-R	AATCACCCTTGCTTCCCTTC

Ripk1-F	AGGATGTGGCAAGTTTAAAGAAAG
Ripk1-R	CTGCTTGGAGGTAAGGGCAC
Ripk1-F	AAAGGAATCAGGGAGATGGAAG
Ripk1-R	AGTTCTCGGTTGTACTGACATG
IL-18-F	TGGAATCAGACCACTTTGGCA
IL-18-R	TCTGGGATTCGTTGGCTGTT
Bak1-F	GCCTACGAACTCTTCACCAAG
Bak1-R	AGGAAGCCAGTCAAACCAC
Bax-F	GGCGAATTGGAGATGAACTG
Bax-R	CCCCAGTTGAAGTTGCCAT
Sting-F	CTGGACCTTCAGAGCTTGGC
Sting-R	CTGGCAAGATCAGCTTCAGGT

Table S3. Information of antibodies applied in this study

Name	Manufacturer	Diluation
Nlrp3	invitrogen #PA5-79740	1:1000
MUC20	ThermoFisher #PA5-50238	1:1000 for WB;1:200 for IF
WT-1	santa cruz #sc-7385	1:1000
LAMP1	proteintech #21997-1-AP	1:1000 for WB;1:200 for IF
TBK1	CST #3504T	1:1000
Phospho-TBK1	CST #5483T	1:1000
Phospho-IRF-3	affinity # AF2436	1:1000
IRF-3	CST #11904T	1:1000
MEK1/2	CST #8727T	1:1000 for WB;1:200 for IF
STING	proteintech # 19851-1-AP	1:1000 for WB;1:200 for IF
phospho-STING	affinity # AF7416	1:1000
Cleaved-Caspase 1	affinity # AF4005	1:1000
Phospho-Met	CST # 3077T	1:1000
Met	CST # 3127S	1:1000
Phospho-MEK1/2	CST # 9154T	1:1000
Anti-CD146	abcam # ab75769	1:1000
MUC4	santa curz #sc-33654	1:100
MUC1	(santa cruz #sc-53381	1:500 for WB; 1:100 for IF
ERp72	CST #503T	1:1000
GAPDH	Proteintech #60004-1-Ig	1:1000
HRP conjugated Anti-rabbit IgG (H + L)	Beyotime #A0208	1:10000
HRP conjugated Anti-mouse IgG (H + L)	proteintech #SA00001-1	1:10000
Fluorescein labeled LTL	Vector #FL-1321	1:500
AQP2	Santa Cruz #sc-9882	1:200
Fluorescein labeled DBA	Vector #FL-1031-5	1:500
Fluorescein labeled PNA	Vector # FL-1071-5	1:500
